# A predictive model for the ichnological suitability of the Jezero crater, Mars: searching for fossilized traces of life-substrate interactions in the 2020 Rover Mission Landing Site

**DOI:** 10.7717/peerj.11784

**Published:** 2021-09-23

**Authors:** Andrea Baucon, Carlos Neto de Carvalho, Antonino Briguglio, Michele Piazza, Fabrizio Felletti

**Affiliations:** 1 DISTAV, University of Genoa, Genova, Italy; 2 Geology Office of Idanha-a-Nova, Naturtejo UNESCO Global Geopark, Idanha-a-Nova, Portugal; 3 Instituto D. Luiz, Faculdade de Ciências da Universidade de Lisboa, University of Lisbon, Lisbon, Portugal; 4 Dipartimento di Scienze della Terra ‘Ardito Desio’, University of Milan, Milan, Italy

**Keywords:** Paleontology, Ichnology, Bioturbation, Bioerosion, Biostratification, Ichnofossil, GIS, Predictive modelling, Mars, Astrobiology

## Abstract

Ichnofossils, the fossilized products of life-substrate interactions, are among the most abundant biosignatures on Earth and therefore they may provide scientific evidence of potential life that may have existed on Mars. Ichnofossils offer unique advantages in the search for extraterrestrial life, including the fact that they are resilient to processes that obliterate other evidence for past life, such as body fossils, as well as chemical and isotopic biosignatures. The goal of this paper is evaluating the suitability of the Mars 2020 Landing Site for ichnofossils. To this goal, we apply palaeontological predictive modelling, a technique used to forecast the location of fossil sites in uninvestigated areas on Earth. Accordingly, a geographic information system (GIS) of the landing site is developed. Each layer of the GIS maps the suitability for one or more ichnofossil types (bioturbation, bioerosion, biostratification structures) based on an assessment of a single attribute (suitability factor) of the Martian environment. Suitability criteria have been selected among the environmental attributes that control ichnofossil abundance and preservation in 18 reference sites on Earth. The goal of this research is delivered through three predictive maps showing which areas of the Mars 2020 Landing Site are more likely to preserve potential ichnofossils. On the basis of these maps, an ichnological strategy for the Perseverance rover is identified, indicating (1) 10 sites on Mars with high suitability for bioturbation, bioerosion and biostratification ichnofossils, (2) the ichnofossil types, if any, that are more likely to be present at each site, (3) the most efficient observation strategy for detecting eventual ichnofossils. The predictive maps and the ichnological strategy can be easily integrated in the existing plans for the exploration of the Jezero crater, realizing benefits in life-search efficiency and cost-reduction.

## Introduction

Seeking signs of past life (biosignatures *sensu* Slater, 2009; Gargaud, 2011) in the geological record of Mars is one of the four primary goals of the NASA Mars 2020 mission (Mustard et al., 2013a; Manrique et al., 2020; NASA, 2020a). To this aim, the mission payload includes a robotic rover, Perseverance, which was launched from Earth on July 30th, 2020 (Maki et al., 2020). The mission landed on February 18th, 2021 on Jezero Crater, an impact crater that is located in the NE region of Mars (Shahrzad et al., 2019; Horgan et al., 2020; Maki et al., 2020).

**Figure 1:**
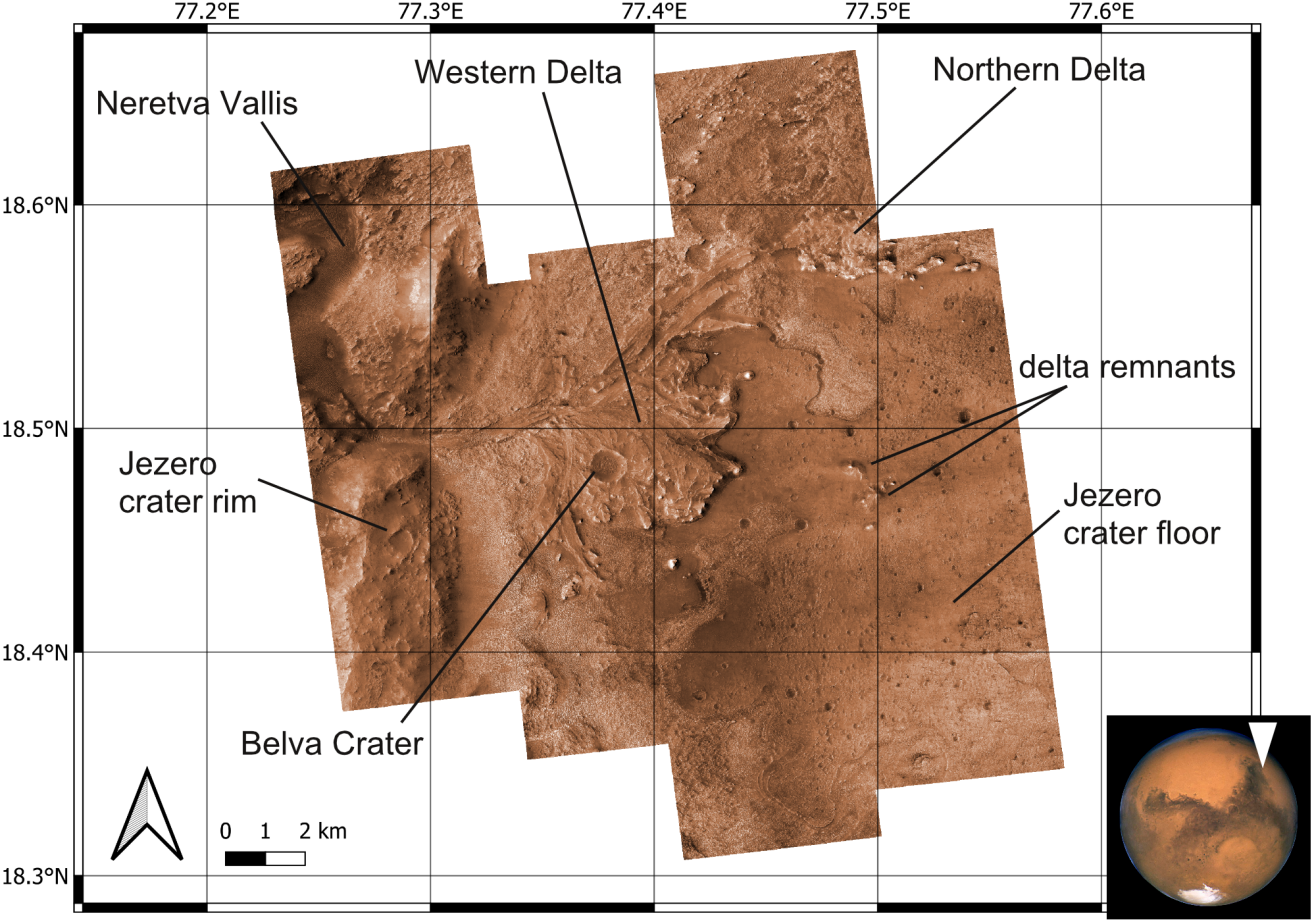
Study area. The basemap has been obtained by colourizing the HiRISE visible map.

The detection of potentially life-supporting (habitable) palaeoenvironments and the identification of deposits with high potential to preserve possible biosignatures have been key aspects of landing site selection (Grant et al., 2018; Mangold et al., 2020). The fact that the Jezero crater hosted a palaeolake with two deltas, as well as inlet and outlet valleys, is one of the major reasons why it has been selected as the landing site for the Perseverance rover (Mangold et al., 2020) (Fig. 1). Evidence of current or past water is regarded as a key requirement for habitability because liquid water is required by all organisms on Earth (Tosca, Knoll & McLennan, 2008). Depositional environments dominated by hydrodynamically quiet, fine-grained sedimentation, such as the deltaic bottomsets within the Jezero crater, have a high concentration and preservation potential for organic matter (Summons et al., 2011; Mangold et al., 2020). The presence of lacustrine carbonates throughout the region and inside the Jezero crater make this palaeolake a landing site of great interest not only for in-situ studies but also for potential sample return (Ehlmann et al., 2008a; Ehlmann et al., 2008b; Mangold et al., 2020). In fact, lacustrine carbonates have a high potential of preserving morphologic, organic, and isotopic biosignatures (Berra, Felletti & Tessarollo, 2019; Horgan et al., 2020).

The Perseverance payload includes several tools for detecting biosignatures (Williford et al., 2018). The SuperCam tool is a suite of four co-aligned instruments that allows detection of morphological biosignatures and organics on a broad survey scale using remote Raman, fluorescence, high-resolution micro-imaging and VISIR spectroscopy (Maurice et al., 2015). For instance, SuperCam will allow the identification of coatings and their possible relationship to biological activity and characterize the regolith potential for biosignature preservation (Maurice et al., 2015). Arm-mounted tools (PIXL, SHERLOC) will perform finer scale observations. Organics (*e.g.*, hopanes, steranes, organic macromolecules) will be searched using SHERLOC, a Deep UV native fluorescence and resonance Raman spectrometer (Beegle et al., 2015). WATSON, based on the Mars Hand Lens Imager, has been added to the instrument, allowing fine-scale colour imaging of rock samples (Martin et al., 2020). The presence of morphological and chemical biosignatures will be investigated with PIXL, a micro-focus X-ray fluorescence spectrometer. It can reveal spatial variations in morphology and chemistry at hand lens-scale view, allowing the detection of (eventual) stromatolite laminations (Allwood et al., 2020).

**Figure 2:**
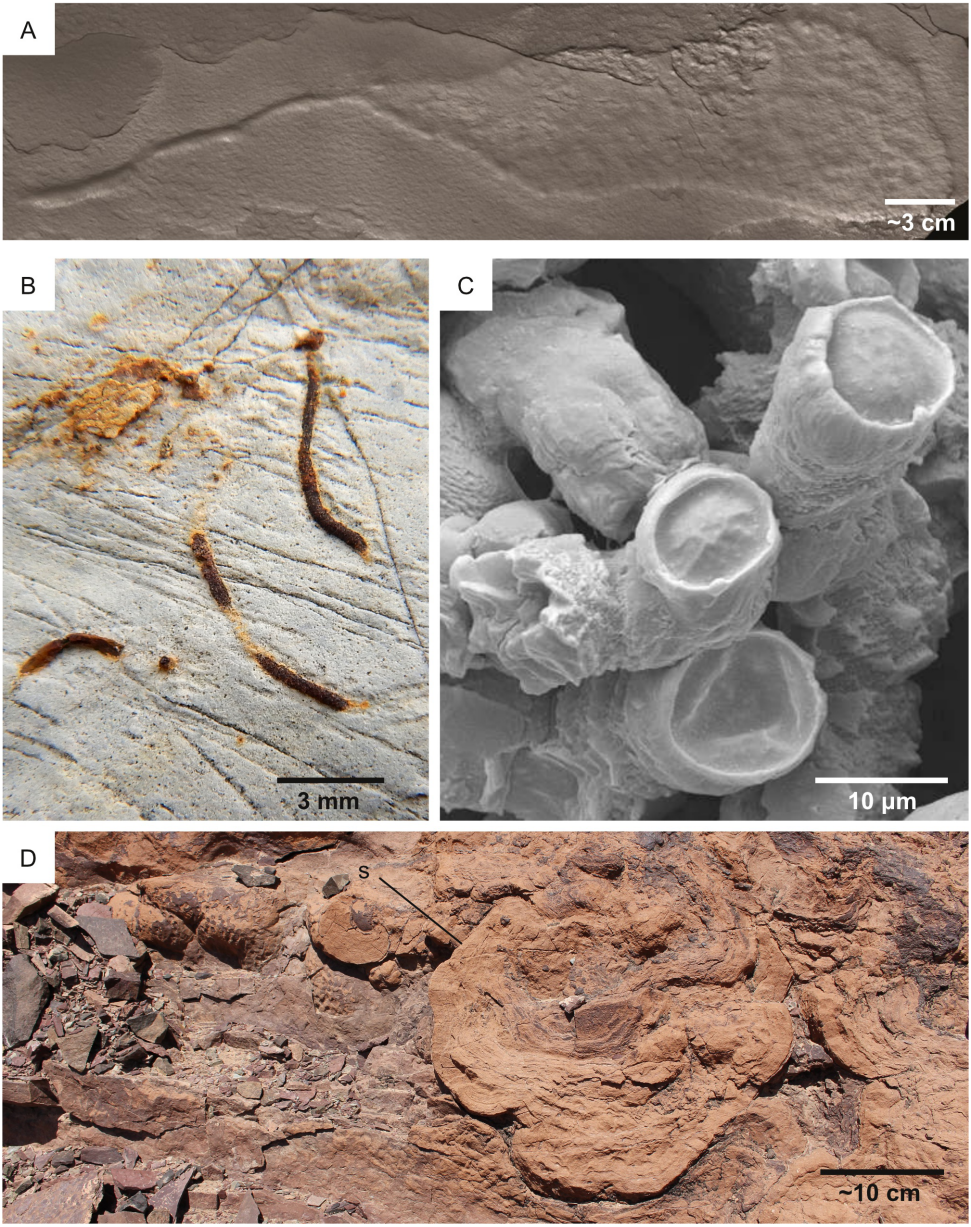
Ichnofossil types on which the predictive model is focused. Each of the figured ichnofossils has been produced by microbes, including eukaryotes (A) and prokaryotes (B–D). (A) Bioturbation ichnofossil: 2.1 Ga locomotion trace attributed to the activity of mobile cell aggregates comparable to cellular slime molds. Image from El Albani et al. (2019). (B) Bioturbation ichnofossil: the vertical burrow *Trichichnus,* attributed to the activity of sulfide-oxidizing bacteria. Pliocene, Ventimiglia (Italy). (C) Bioerosion ichnofossil: *Fascichnus,* produced by boring bacteria (possibly the cyanobacterium *Hyella caespitosa*)*.* Scanning electron microscopy image of a 3-dimensional epoxy cast of the microborings. Carboniferous, Renox Creek (U.S.A.). The image is from Hannon & Meyer (2014). (D) Biostratification ichnofossil: a stromatolite from the Lower Cambrian Lalun Formation of the Tabas area (Iran).

The resolution of the Perseverance tools allows imaging of (potential) products of life-substrate interactions, such as burrows, borings, trails, stromatolites and microbial-induced sedimentary structures (MISS). Nevertheless, their study (ichnology) received little attention in astrobiology (see the review of Baucon et al., 2017) and almost no attention in the context of the Mars 2020 mission. For instance, the PIXL and SuperCam tools can image eventual macroscopic and microscopic products of bioturbation, *i.e.,* the process by which the primary consistency and structure of sediment are modified by the activities of organisms living within it (Frey & Pemberton, 1985; Bromley, 1996; Pemberton et al., 2001) (Figs. 2A, 2B). However, the Mars 2020 documents (Mustard et al., 2013b; Hays et al., 2017) do not include bioturbation structures among the biosignatures to be searched on Mars. Bioerosion, the mechanical or biochemical drilling of a rigid substrate (Fig. 2C) (Frey & Pemberton, 1985; Bromley, 1996; Pemberton et al., 2001), is rarely mentioned in research related to Mars 2020 (*e.g.*, Czaja et al., 2020; Ivarsson, Sallstedt & Carlsson, 2020). Biostratification, *i.e.,* the process by which organisms impart stratification features to the substrate (Fig. 2D), can result in microbialites such as MISS and stromatolites, which are the only product of life-substrate interactions that received thorough attention in the context of the Mars 2020 mission (Mustard et al., 2013b; Hays et al., 2017). According to the Landing Site Data Sheets (NASA, 2020b), microbial-induced sedimentary structures may have been preserved in the quiet deltaic or lacustrine deposits of the Jezero Crater.

This lack of attention is surprising because plausible ancient Martian biosignatures are considered to be similar to the types of biosignatures characterizing the Precambrian rock record of Earth (McMahon et al., 2018; Czaja et al., 2020), which is indeed rich in fossilized products of life-substrate interactions (ichnofossils). The Precambrian rock record comprises 1.7 Ga (billion years) microborings (Zhang & Golubic, 1987), 2.1 Ga macroscopic burrows (El Albani et al., 2019), 3.2 Ga MISS (Noffke et al., 2006; Heubeck, 2009; Noffke, 2009) and 3.49 Ga stromatolites (Allwood et al., 2007). The abundant Precambrian ichnofossil record is related to the excellent preservation potential of ichnofossils, which often record the activity of soft-bodied organisms that usually do not fossilize. Ichnofossils are resilient to processes (*e.g.*, mechanical and chemical degradation, diagenesis, tectonism, metamorphism and meteorite impact) that obliterate other biosignatures, such as body fossils, as well as chemical and isotopic evidence for past life (Baucon et al., 2017).

The occurrence of bioturbation, bioerosion and biostratification ichnofossils within ancient Earth deposits, even when putative, encourages the application of ichnological studies to the Mars 2020 mission.

The ichnological approach is further supported by the presence of corresponding ichnofossil-like structures on Mars. Elongate structures, with sudden changes in orientation, resembling bioturbation ichnofossils have been reported from the Vera Rubin Ridge, in the Eastern part of Mars (Baucon et al., 2020a). Microboring-like structures, consisting of curved and dendritic microtunnels, have been observed in the Martian meteorites Nakhla and Yamato 000593 (Fisk et al., 2006; Gibson et al., 2006; McKay et al., 2006; White et al., 2014). Structures possibly related to biostratification have been reported from the <3.7 Ga Gillespie Lake Member on Mars (Noffke, 2015). Although the biogenicity of these ichnofossil-like structures from Mars is highly debated, they inform about the feasibility of the ichnological approach to the Mars 2020 mission.

The goal of this paper is to fill this methodological gap by evaluating the suitability of the Mars 2020 Landing Site for ichnofossils. To this goal, this works applies palaeontological predictive modelling, a technique used to predict the location of fossil sites in uninvestigated areas on Earth (Oheim, 2007; Anemone, Emerson & Conroy, 2011). Before of palaeontological application, predictive modelling has been widely used by archaeologists to find new sites and to identify areas in greatest need of protection (Kohler & Parker, 1986; Mehrer & Wescott, 2005; Oheim, 2007; Verhagen, 2018). Predictive modelling assumes that palaeontological sites are not randomly distributed but their location is related to certain characteristics of the modern and past environment, *e.g.*, percent of bedrock covered by vegetation, the permanence of water, and ancient oxygen levels (Oheim, 2007; Verhagen, 2007; Anemone, Emerson & Conroy, 2011). Such characteristics are typically ranked and combined to produce a predictive map, *i.e.,* a raster map of cells (pixels) where each cell contains a probability value representing the potential of containing a palaeontological site (*e.g.*, Oheim, 2007; Anemone, Emerson & Conroy, 2011). In parallel to these applications, the goal of this research will be delivered through a set of predictive maps showing which areas of the Mars 2020 landing site in the Jezero crater are more likely to preserve ichnofossils. The predictive nature of this study should be highlighted, *i.e.,* the predictive model aims at detecting areas of high ichnological potential on Mars, but this does not necessarily imply the existence of life on Mars. Accordingly, predictive modelling can be used as a scientific tool to guide future efforts to the most ichnologically sensitive regions of Jezero crater, realizing benefits in life-search efficiency and cost-reduction.

### Geological setting

Jezero is a 45-km wide impact crater in the north-eastern area of Mars in the Syrtis Major quadrangle (18.2°N, 77.6°E), a region dominated by a mafic crust (Horgan et al., 2020; Mangold et al., 2020). The central basin floor of the Jezero crater is capped by a ∼13 m thick volcanic unit, whereas sedimentary deposits are observed close to the crater rim (Schon, Head & Fassett, 2012; Shahrzad et al., 2019). Based on crater size-frequency distribution, the volcanic unit has been dated back to the Early Amazonian (Schon, Head & Fassett, 2012; Shahrzad et al., 2019). Two ancient fluvial valleys enter into the Jezero crater, Neretva Vallis to its west (Fig. 1) and an unnamed valley to the north (Fassett & Head, 2005; Mangold et al., 2020). Deltaic deposits are found at the mouth of the corresponding palaeorivers within the Jezero crater. The Western Delta is dominated by Fe/Mg smectites and exhibits well defined sedimentary layering, whereas the Northern Delta is dominated by Mg-carbonates and associated olivine, but is less well preserved (NASA, 2020b). The delta plain environment is the most well-preserved depositional setting of the Jezero delta complex, whereas most of the prodelta deposits have been eroded by aeolian processes (Schon, Head & Fassett, 2012; Day & Dorn, 2019). Accordingly, the present front of the Jezero fan is not a primary depositional feature but a steep (≥10–30°) erosional escarpment (Schon, Head & Fassett, 2012). Isolated distal remnants of sedimentary material, located ∼3 km from the continuous deposit, rise ∼150 m above the basin floor and also serve as indicators of the larger previous extent of the delta (Schon, Head & Fassett, 2012). Jezero crater is the only known location on Mars where orbital detections of carbonates are found close to robust fluvio-lacustrine features (Horgan et al., 2020). Jezero crater has been studied for more than a decade, but the timing and duration of its fluvial and lacustrine activity are still poorly constrained (Mangold et al., 2020). According to Fassett & Head (2008), incision of the Jezero valley system ended at approximately 3.8 Ga, at the Noachian–Hesperian boundary (see also Goudge et al., 2018).

This sedimentologic, stratigraphic and geomorphic evidence allowed to reconstitute to a certain extent the palaeoenvironmental evolution of the Jezero crater. Accommodation space resulted from the formation of a Noachian-aged impact crater (Schon, Head & Fassett, 2012; Goudge et al., 2018). Successively, the Jezero crater rim was breached by crater degradation processes and precipitation-fed valley networks, initiating the filling of the basin (Schon, Head & Fassett, 2012). Formation of the outlet channel began once these valley networks flooded the crater basin (Schon, Head & Fassett, 2012). Although the Jezero delta is thought to have formed when a river flowed into the Jezero crater around the Late Noachian/Early Hesperian boundary (Fassett & Head, 2008; Schon, Head & Fassett, 2012), its age remains uncertain, and the duration of surface flows that formed the delta is poorly constrained (Lapôtre & Ielpi, 2020). It is hypothesized that the majority of Martian fluvial activity peaked at approximately the Noachian-Hesperian boundary and ceased shortly thereafter (Goudge et al., 2015). The carbonate unit may represent authigenic lacustrine carbonates, precipitated in the near-shore environment of the Jezero palaeolake (Horgan et al., 2020). The presence of significant residual accommodation space in the Jezero Crater indicates that sediment transport and deposition into the lake terminated before the basin was completely filled (Schon, Head & Fassett, 2012). Duration estimates for delta deposition and lake persistence vary from several years to millions of years (Schon, Head & Fassett, 2012; Goudge et al., 2015; Mangold et al., 2020).

## Materials & Methods

### GIS organization

**Figure 3:**
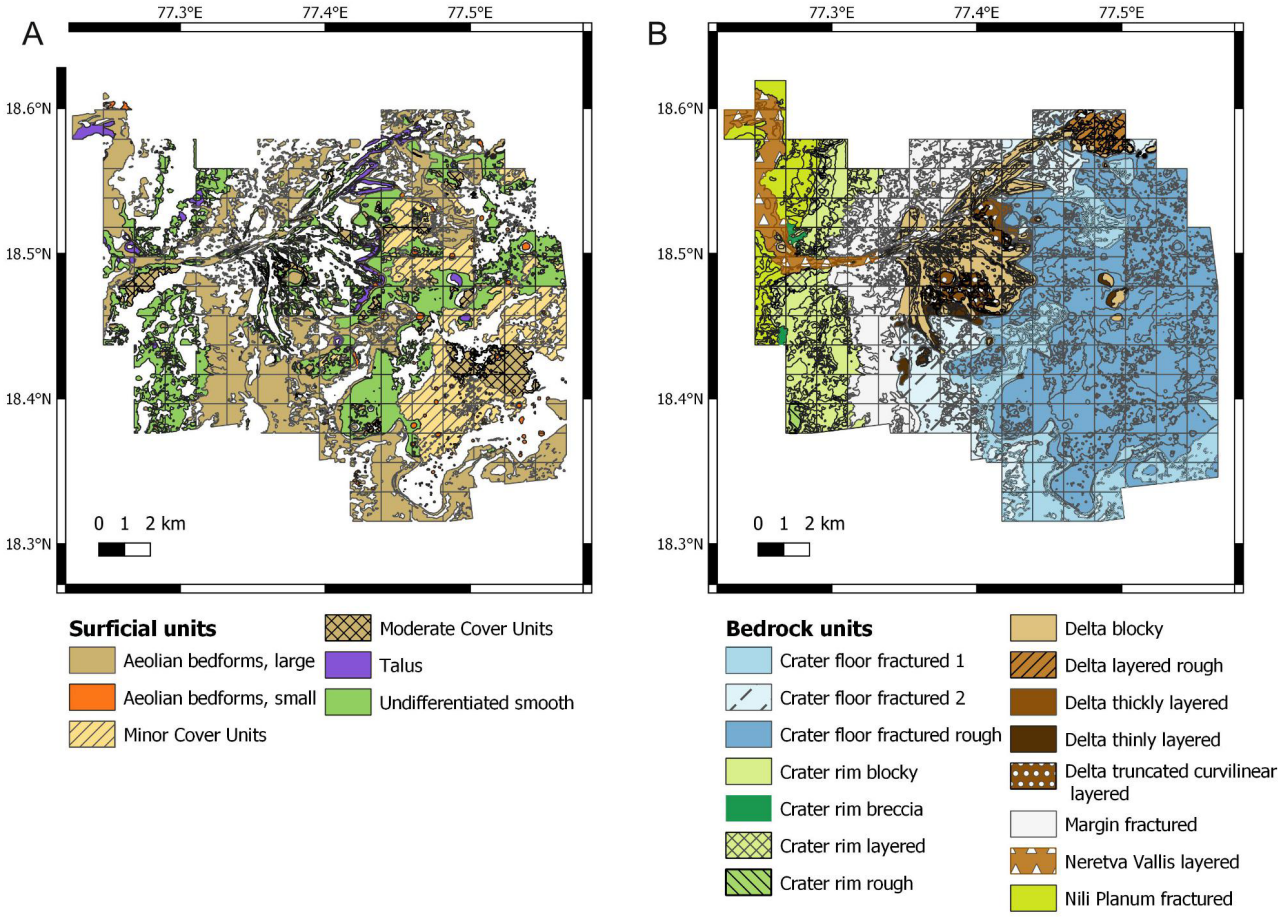
Raw data used for establishing the suitability scores. Data derive from the photogeologic map of Stack et al. (2020). (A) Map of the surficial units. (B) Map of the bedrock units.

This study applies predictive modeling, a technique used in palaeontology and archaeology (Oheim, 2007; Anemone, Emerson & Conroy, 2011), to predict the location of (eventual) ichnofossil sites in the Mars 2020 Landing Site. Predictive modelling typically uses a geographic information system software to combine attributes associated with the preservation and distribution of fossils (Oheim, 2007). In this study, the software QGIS 3.10.12 ‘A Coruña’ (QGIS.org, 2021) is used to develop a geographic information system of the Mars 2020 Landing Site. The extent of the study area corresponds to the area of greatest scientific interest to the Mars 2020 Science Team and where high-resolution data of the High Resolution Imaging Science Experiment (HiRISE) are available (Stack et al., 2020). The map of Stack et al. (2020) (Fig. 3) is also used for deriving palaeoenvironmental information about Mars.

The GIS of the study area is organized in six input layers and three output predictive layers (Table 1). Each input layer maps the suitability for one or more ichnofossil types (bioturbation, bioerosion, biostratification structure) based on the assessment of a single attribute (suitability factor) of the Martian environment. In other words, any location within a single input layer is associated with a suitability score about the most desirable or least desirable conditions relative to ichnological site location. As a result, each location of the Mars 2020 Landing Site is associated with six suitability scores (Table 2). We followed the predictive modelling procedure of Oheim (2007), which successfully used four levels of classification for suitability scores. Accordingly, in this study suitability scores range from 1 to 4, with 4 representing the most desirable conditions (*e.g.*, uncovered bedrock) and 1 the least desirable ones (*e.g.*, completely covered bedrock). The scoring system is a relative one, *i.e.,* it informs about how a location of the study area ranks in relation to the others. Scores are attributed based on the characteristics of the geological units (Table 3) of the Mars 2020 landing site.

**Table 1:** Layer organization of the GIS.

	Layer name	Mapped suitability score	Score variable	Suitability factor assessed
Input layers	1 - Bioturbation_score_substrate	Substrate suitability for bioturbation	*L*	Presence of loose substrates
	2 - Bioerosion_score_substrate	Substrate suitability for bioerosion	*H*	Presence of lithified substrates (hardgrounds)
	3 - Energy_score	Suitability of the energy regime for ichnofossils	*E*	Kinetic energy level (*e.g.*, hydrodynamic energy)
	4 - Sedimentation_score	Suitability of the sedimentation rate for ichnofossils	*S*	Sedimentation rate
	5 - Water_score	Suitability of the water table level for ichnofossils	*W*	Presence of a permanent body of water
	6 - Cover_score	Suitability of the cover conditions for ichnofossils	*K*	Presence of unconsolidated deposits that cover bedrock
Predictive layers	7 - Bioturbation_map	Suitability for bioturbation structures	*A*	–
	8 - Bioerosion_map	Suitability for bioerosion structures	*B*	–
	9 - Biostratification_map	Suitability for biostratification structures	*C*	–

**Table 2:** Suitability scores attributed to the geological units of the Mars 2020 landing site (*sensu*Stack et al., 2020). The characteristics of the geologic units are summarized in Table 3. The *W* score is not reported because it is based on the position of the water table, therefore it is based on elevation.

	Unit name	*L*	*H*	*E*	*S*	*K*
Bedrock units	Crater floor fractured 1	4	2	4	4	–
	Crater floor fractured 2	4	2	3	3	–
	Crater floor fractured rough	4	2	4	4	–
	Margin fractured	4	2	3	2	–
	Crater rim blocky	1	4	2	4	–
	Crater rim breccia	1	4	1	4	–
	Crater rim layered	1	4	2	4	–
	Crater rim rough	1	4	2	4	–
	Neretva Vallis layered	4	1	1	3	–
	Nili Planum fractured	1	4	2	4	–
	Delta blocky	4	1	2	1	–
	Delta layered rough	4	1	4	4	–
	Delta thickly layered	4	1	4	4	–
	Delta thinly layered	4	1	4	4	–
	Delta truncated curvilinear layered	4	1	3	3	–
Surficial units	Aeolian bedforms, large	–	–	–	–	1
	Aeolian bedforms, small	–	–	–	–	2
	Minor Cover Units	–	–	–	–	2
	Moderate Cover Units	–	–	–	–	1
	Talus	–	–	–	–	3
	Undifferentiated smooth	–	–	–	–	2

**Table 3:** Geological units of the Mars 2020 landing site. Based on Stack et al. (2020) and references therein. The spatial distribution of the units is shown in Fig. 4.

	Unit name	Tone	Layered	Texture	Remarks	Interpretation
Bedrock units	Crater floor fractured 1	Light	No	fractured and blocky	Massive	Unspecific tephra, airfall, aeolian or lacustrine deposit
	Crater floor fractured 2	Light	No	Rough and fractured		Unspecific tephra, airfall, aeolian or lacustrine deposit
	Crater floor fractured rough	Light to medium	No	Rough, boulder-producing	Highly crater-retaining	Unspecific tephra, airfall, aeolian or lacustrine deposit
	Margin fractured	Light	No	Fractured		Unspecified tephra or marinal lacustrine
	Crater rim blocky	Intermediate	No	Rubbly, massive	Forming high-standing ridges that erode into boulders	Pre-impact basement bedrock of unspecified sedimentary or volcanic origin
	Crater rim breccia	Light and intermediate	No	Brecciated and disrupted	exposed on the Nili-Planum facing slope of the Jezero crater rim	Syn-Isisdis or syn-Jezero impact breccia
	Crater rim layered	Light	Yes	Polygonally fractured	occasionally faulted and distrupted	Pre-impact basement bedrock of unspecified sedimentary or volcanic origin
	Crater rim rough	Light	No	Rough texture, polygonally fractured	high crater retention	Unspecified clastic sedimentary or explosive volcanic deposit
	Neretva Vallis layered	Light to intermediate	Yes	Polygonally fractured	Layered outcrops exhibiting m-scale polygonal fractures	Fluvial deposits
	Nili Planum fractured	Light	No	Rough, fractured		Unspecified tephra, airfall or aeolian
	Delta blocky	Intermediate	No	Blocky	blocks of variable tone	Coarse-grained fluvial channel deposit
	Delta layered rough	Light	Yes	Rough, mottled	Parallel, m-thick layers	Distal deltaic deposits
	Delta thickly layered	Light	Yes	Rough	Erosionally resistant layers up to several meters thick	–
	Delta thinly layered	Alternating light and dark bands	Yes	Polygonally fractured		deltaic or lacustrine deposit; delta plain; prodelta; hemipelagic
	Delta truncated curvilinear layered	Alternating light and dark bands	Yes	Curvilinear sets of alternating layers	Sets truncate one against another	Laterally accreting point bars or subaqueous channel-levee complexes
Surficial units	Aeolian bedforms, large	Light	No	–	aeolian bedroms	Transverse aeolian ridges
	Aeolian bedforms, small	Dark	No	–	straight-crested bedforms Reticulate pattern common	Wind ripples
	Minor Cover Units	–	No	–	areas with partial cover for which differentiating bedrock from surficial deposit at map scale (>0 to ∼ 25% cover)	–
	Moderate Cover Units	–	No	–	Areas with partial cover for which differentiating bedrock from surficial deposit at map scale (∼ 25–75% cover)	–
	Talus	–	No	–	Boulder accumlations	Blocks eroded from the bedrock via weathering
	Undifferentiated smooth	dark	No	–	Smooth deposits draping topography	Unconsolidated mantling deposits (dust, sand, pebbles, cobbles)

**Figure 4:**
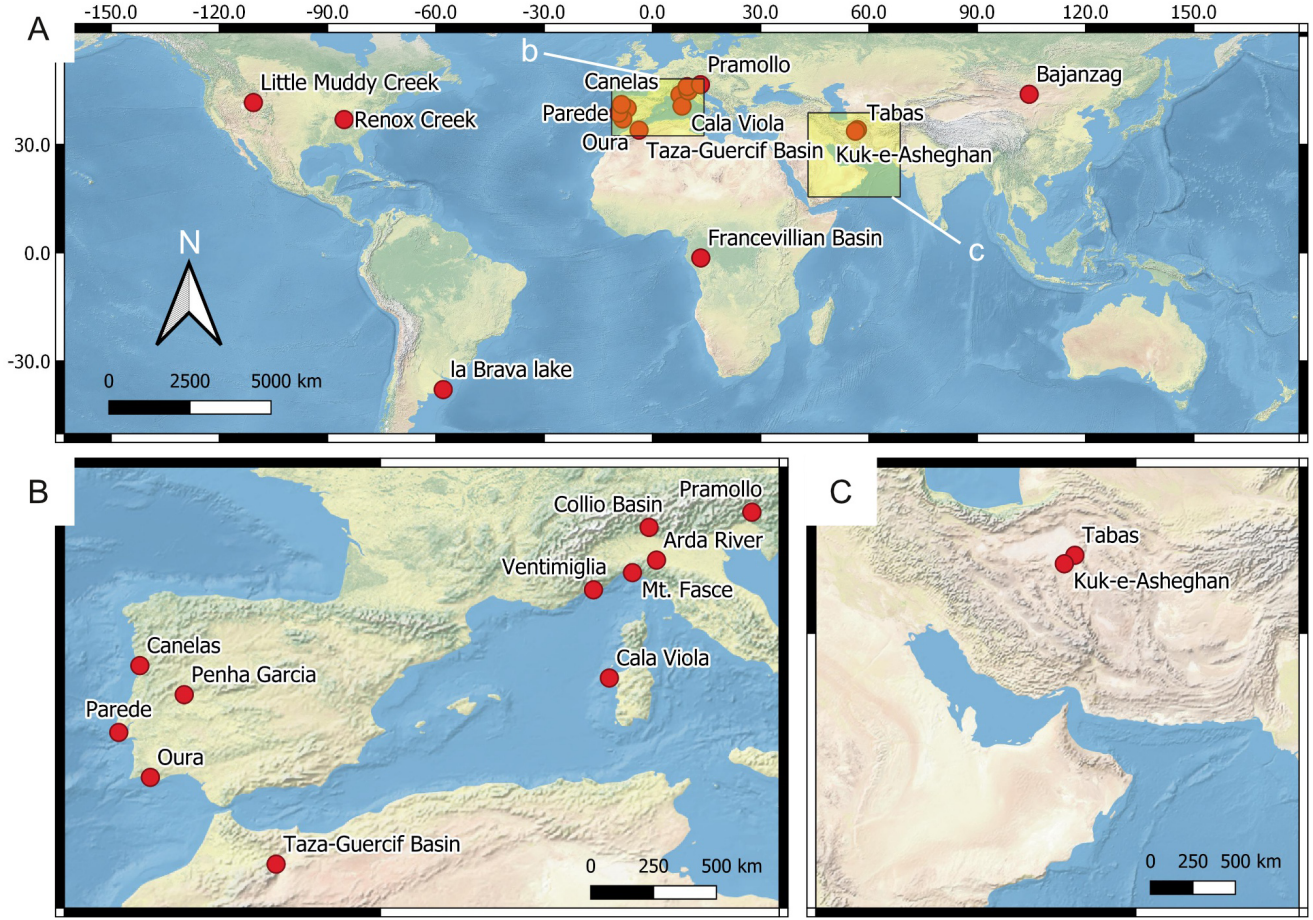
Location of the reference ichnosites. (A) Global overview of the ichnosites discussed in this study. The insets b and c show the area detailed in B and C, respectively. (B) European and North African sites. (C) Western Asia sites.

The predictive layers result from the weighted overlay of multiple input layers (Table 1). The predictive layers map the suitability for a specific ichnofossil type, *e.g.*, the higher the value on the bioturbation map layer, the more suitable the corresponding location it is for the preservation of bioturbation ichnofossils.

### Source data collection

The source data for constructing the predictive model comprise (1) ichnosite data, (2) vector data, and (3) raster data. 18 reference ichnosites on Earth are considered for assessing suitability scores and the importance (weight) of each suitability factor in controlling ichnofossil distribution. The reference ichnosites are selected because of their similarity with the Mars 2020 Landing Site in terms of depositional environment (fluvial, lacustrine), processes (deltaic processes), genetic surfaces (unconformities) or exploration conditions. Location, palaeoenvironment and age of the ichnosites are presented in Fig. 4 and Table 4. We conducted fieldwork in each of the reference ichnosites, with few exceptions. Specifically, four sites (Renox Creek, La Brava Lake, Little Muddy Creek and the Francevillian Basin) have been studied using bibliographic references (Hannon & Meyer, 2014; El Albani et al., 2014; El Albani et al., 2019; Tietze & Esquius, 2018; Zonneveld, Bartels & Clyde, 2003). During fieldwork, ichnofossils have been photographed using different cameras: Nikon Coolpix W300, Canon EOS 100D, Sony DSC-HX60V, Fujifilm FinePix S9500, Olympus X450. The specifications of these cameras are comparable to those of the imaging tools mounted on the Perseverance rover, thus allowing to test the feasibility of the ichnological approach on Mars. Specifically, the field of view, resolution, and focal length of the fieldwork cameras are within the range of the Perseverance imaging tools, as defined in Mars 2020 technical reports and analogue studies (Godin, Caudill & Osinski, 2017; Edgett, Caplinger & Ravine, 2019; Martin et al., 2020).

**Table 4:** Reference ichnosites. ‘fieldwork’ refers to the ichnosites that have been investigated on the field during the development of this study; conversely, ‘literature’ indicates ichnosites that have been studied using bibliographic references.

Site	Country	Age	Palaeoenvironment	Research approach	References
La Brava lake	Argentina	Modern	lacustrine	literature	Tietze & Esquius (2018)
Arda River	Italy	Pleistocene	deltaic (marine), shallow-marine	fieldwork	Crippa et al. (2018)
Ventimiglia	Italy	Pliocene	deltaic (marine)	fieldwork	Breda, Mellere & Massari (2007)
Oura	Portugal	Miocene	shoreface	fieldwork	Cachão et al. (2009)
Taza-Guercif Basin	Morocco	Miocene	deep-marine (channel-levee system)	fieldwork	Felletti et al. (2020)
Little Muddy Creek	U.S.A.	Eocene	fluvial-lacustrine with braid delta	literature	Zonneveld, Bartels & Clyde (2003)
Mt. Fasce	Italy	Cretaceous	deep-marine	fieldwork	Uchman (2007)
Bayanzag	Mongolia	Cretaceous	fluvial	fieldwork	Loope & Dingus (1999)
Parede	Portugal	Cretaceous	carbonate shoreface and tidal flat	fieldwork	Santos et al. (2015)
Cala Viola	Italy	Permian-Triassic	fluvial, aeolian	fieldwork	Baucon et al. (2014)
Collio Basin	Italy	Permian	fluvial-lacustrine with carbonate deposition	fieldwork	Berra & Felletti (2011) Berra, Felletti & Tessarollo (2016)
Pramollo Basin	Italy-Austria	Carboniferous (Pennsylvanian) -Permian	fluvio-deltaic	fieldwork	Baucon & Neto de Carvalho (2008), Baucon et al. (2015a), Baucon et al. (2015b)
Renox Creek	U.S.A.	Carboniferous (Mississippian)	deltaic-influenced marine (50 km from the delta front)	literature	Hannon & Meyer (2014)
Canelas	Portugal	Ordovician	offshore	fieldwork	Neto de Carvalho et al. (2016)
Penha Garcia	Portugal	Ordovician	deltaic (marine), shallow marine	fieldwork	Neto de Carvalho, Rodrigues & Baucon (2014) and Neto de Carvalho et al. (2016)
Kuk-e-Asheghan	Iran	Ordovician	nearshore, shelf	fieldwork	Bayet-Goll et al. (2016)
Tabas area	Iran	Cambrian	fluvio-deltaic, nearshore	fieldwork	Bayet-Goll, Geyer & Daraei (2018)
Francevillian Basin	Gabon	Palaeoproterozoic	shallow-marine (overlying deltaic deposits)	literature	El Albani et al. (2014) and El Albani et al. (2019)

Vector and raster data are used to evaluate the distribution of environmental parameters across the Mars 2020 landing site. Vector data comprise the shapefile of the photogeologic map of the Perseverance rover field site Team (Stack et al., 2020). Raster data include HiRISE image pairs (NASA, 2020c), the HiRISE visible base map (USGS Astrogeology Science Center, 2020a), and the HiRISE digital terrain model (USGS Astrogeology Science Center, 2020b).

### Workflow

The predictive model of the Mars 2020 landing site is obtained by following a five-step workflow. The procedure is based on the predictive modelling workflow of Balla et al. (2014), which has been slightly modified to accommodate the lack of field data from the Jezero crater. The following steps have been applied:

1. Selection of the suitability factors: selecting the environmental parameters (suitability factors) controlling the distribution of potential ichnofossils;
2. Proxy assessment: determining the geological proxies that inform on the suitability factors selected in step 1;
3. Quantification of the suitability scores: attributing suitability scores to each location of the Mars 2020 Landing Site based on the assessment of suitability factors;
4. Assessment of suitability weights: estimating the suitability weights, namely the importance of each suitability factor in controlling ichnosite location;
5. Data aggregation: adding together the scores of multiple suitability factors (overlay analysis) to identify the most suitable locations for bioturbation, bioerosion and biostratification ichnofossils.

The methodological aspects of the workflow are presented below, whereas the assessment of predictive variables (*e.g.*, suitability factors, proxies, scores and weights) are thoroughly discussed in the next section because of the specificity of the subject. From the methodological perspective, the first step for developing a predictive model requires the selection of the predictive parameters, *i.e.,* the suitability factors that control the distribution of the objects of interest. In fact, predictive models use multi-parametric spatial analysis of georeferenced data to identify areas of possible interest (Store & Kangas, 2001; Balla et al., 2014). In the present study, suitability factors are selected among the environmental attributes that are known to determine ichnosite location on Earth, provided that they are independent from Earth-type life.

Except for the surficial cover, all the selected suitability factors are related to the palaeoenvironmental conditions of Mars, which cannot be directly observed. For this reason, the second step of the workflow requires the identification of environmental proxies, *i.e.,* geological proxies that are informative of the ancient environmental conditions of Mars. This step is based on the principles of (palaeo)environmental analysis, which is the process by which the depositional environment of sediment is determined (Selley, 2000). The characteristics of a depositional environment have a fundamental control on the properties of the resulting rock unit (Nichols, 2009), including texture and sediment size, sedimentary structures, mineralogy and elevation range. In this paper, such characteristics are derived from the HiRISE visible map and the digital terrain model of the landing site, as well as by considering published observations on the Jezero crater (Hoefen, 2003; Ehlmann et al., 2008b; Ody et al., 2013; Goudge et al., 2015; Bramble, Mustard & Salvatore, 2017; Palumbo & Head, 2018; Rogers et al., 2018; Kremer, Mustard & Bramble, 2019; Horgan et al., 2020; Mandon et al., 2020; Stack et al., 2020).

In the third step of the workflow, each location of the study site is attributed a set of suitability scores for ichnofossils. Following the ranking scheme of Oheim (2007), the scores of ichnological suitability range from 1 to 4, with 4 representing the most desirable condition and 1 representing the least desirable condition for ichnofossils. Score assessment is based on theoretical considerations and the characteristics of 18 reference ichnosites on Earth (Table 4).

From the practical side, scores are first attributed to the geological units described in the most recent photogeologic map of the Mars 2020 Landing Site (Stack et al., 2020). Score assessment is based on the environmentally informative characteristics (proxies) of each unit. As a result of the scoring process, four scores are linked to each bedrock unit and a single score is associated to each surficial unit (Table 2). Successively, scores are related to the spatial distribution of each unit, which is derived from the vector file of the photogeologic map of the Mars 2020 Landing Site (Fig. 3). A code snippet is written to automate the process of relating scores to the spatial distribution of the geological units (Supplemental Information 1). The snippet is run using the field calculator of QGIS.

A similar process is followed for the suitability score of the water table position (W). Using the digital terrain model of the landing site, high suitability scores (*W* = 4) are attributed to the locations below the most elevated position of the Jezero lake shoreline (shoreline elevation based on Salese et al., 2020), whereas low suitability scores (*W* = 1) are attributed to those above.

Our predictive model takes into account the fact that some suitability factors are more influential than others in ichnological site distribution. The fourth step is therefore the assessment of the suitability weights, *i.e.,* those suitability factors that have more importance in the model are given a higher percentage influence (weight) than the others. The relative importance of the suitability factors is based on theoretical considerations and observations at the reference sites of Fig. 4.

The fifth step is data aggregation, according to which the scores of multiple suitability factors are weighted and added together to identify the most favourable locations for detecting bioturbation, bioerosion and biostratification ichnofossils. This process is a weighted overlay analysis. Following Balla et al. (2014), aggregation of palaeoenvironmental data is achieved by using Weighted Linear Combination, *i.e.,* each suitability score related to the ancient Martian environment is multiplied with the value of its weight and the results are summed. Provided that the sum of all weights equals to 1, the result will have the same range (1–4) as the one specified for the suitability scores (Balla et al., 2014). Characteristics of the modern Martian environment can preclude the observation of the bedrock, *e.g.*, thick deposits of unconsolidated sediment (surficial cover) can completely obscure the bedrock. To consider this aspect, a quantity describing the surficial cover conditions is subtracted from the result of the aggregation of palaeoenvironmenal data. The following Eq. (1), based on the formula of Balla et al. (2014: p. 122), expresses this aggregation process in a generalized form:


(1) \begin{eqnarray*}N= \left( \sum {x}_{i}{w}_{i} \right) - \left( 4-k \right) \end{eqnarray*}

where *N* is the aggregated suitability score, *x*_*i*_ value of the suitability factor *i*, *w*_*i*_ the weight of the suitability factor *i, k* is the suitability score for the surficial cover. Equation (1) is used to calculate the three aggregated suitability scores of our model, namely the bioturbation (*A*), bioerosion (*B*) and biostratification (*C*) suitability scores (Eqs. (2)–(4)). The same environmental condition can have a different importance for different trace types, therefore, the same suitability factor can be associated with a different weight when calculating *A*, *B*, or *C* (Table 5). To this aim, vector input layers are rasterized and aggregated. The result is a set of three predictive maps, each of which maps the suitability for a specific ichnofossil type (bioturbation, bioerosion or biostratification structure).

**Table 5:** Weights associated with the palaeoenvironmental suitability factors, *i.e*., the suitability of the energy regime (*E*), sedimentation rate (*R*), water table position (*W*) and substrate type (*L*: for bioturbation; *H*: for bioerosion).

Suitability score	Weight for *L*	Weight for *H*	Weight for *E*	Weight for *S*	Weight for *W*	SUM
*A* (suitability for bioturbation structures)	0.5	0	0.1	0.2	0.2	1
*B* (suitability for bioerosion structures)	0	0.5	0.1	0.2	0.2	1
*C* (suitability for biostratification structures)	0	0	0.1	0.4	0.5	1

### Assessment of suitability scores

Many different attributes of the environment, both modern and past, influence fossil preservation and accessibility on Earth (Oheim, 2007), being therefore eligible as suitability criteria for the Mars 2020 predictive model. On Earth, ichnosite location depends on the percentage of surficial cover concealing the bedrock and by the attributes of the palaeoenvironment controlling tracemaker activity, *e.g.*, hydrodynamic energy, substrate cohesiveness, oxygenation, salinity, sedimentation rate, food supply, bathymetry, water turbidity, climate and position of the water table (Bromley, 1996; Buatois & Mángano, 2011; Knaust, 2017). However, only five of these factors are considered in the here proposed model: (1) surficial cover (variable *K*); (2) energy regime (*E*); (3) substrate cohesiveness (variables *L* and *H*); (4) sedimentation rate (*R*); (5) position of the water table (*W*).

These suitability factors were selected because they influence ichnological suitability independently from the planetary locale in which they are found. This criterion for selecting suitability factors is explained by the fact that extraterrestrial ecosystems, if any, may have differed for environmental conditions, biochemistry and evolutionary history from Earth ecosystems (Benner, Ricardo & Carrigan, 2004; McKay, 2010). Even if it is acknowledged that early Earth and Mars shared similar physical and chemical surface properties (Horneck, 2000; Kargel, 2004; Read, Lewis & Mulholland, 2015), their early environmental history was necessarily different, and there cannot be a perfect analogy between the two planets during their early history (Hipkin et al., 2013; Baucon et al., 2017). Also, the evolutionary history of any inhabited astronomical object should be unique (Morris, 1999), and environmental events can drive evolution *via* mass extinctions and directional selection (Schulze-Makuch, Irwin & Fairén, 2013). Factors excluded from the predictive model (*e.g.*, oxygenation, salinity) are closely tied to terrestrial biology, being therefore inappropriate for predicting the ichnological suitability of Martian locations. For instance, ancient oxygenation levels are known to control the distribution of marine ichnofossils on Earth (Bromley & Ekdale, 1984; Baucon et al., 2020b), but this pattern is related to the fact that most metazoan life on Earth evolved to require oxygen (Danovaro et al., 2010). Each of the following sections presents a single selected suitability factor, describing (1) the specific criteria for its selection, (2) the geological proxies used to deduce its spatial variability, (3) the distribution of the related suitability scores.

### Surficial cover

**Figure 5:**
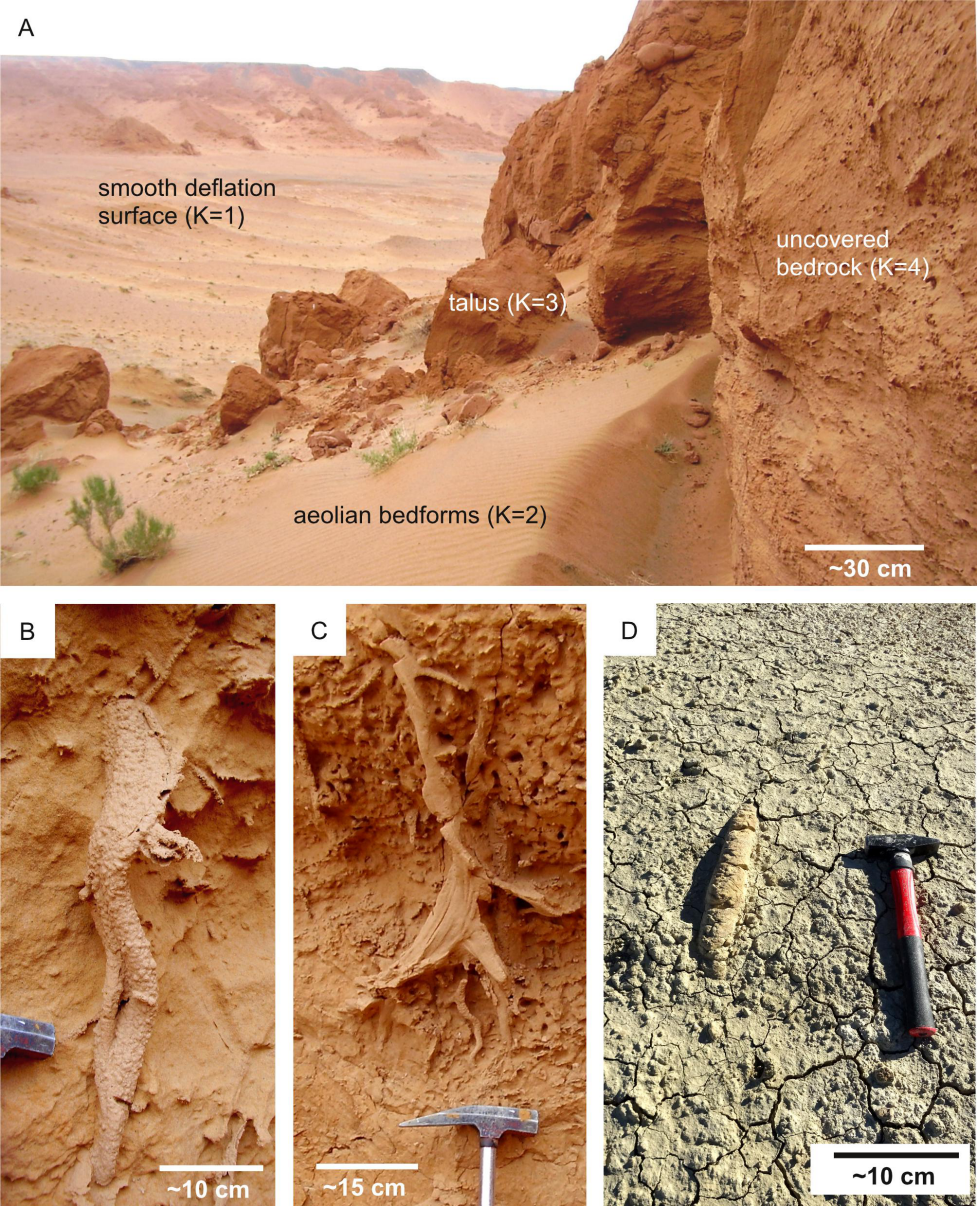
Ichnological suitability of the surficial cover conditions of terrestrial ichnosites. (A) Suitability scores (*K*) of the Bayanzag ichnosite (Cretaceous, Mongolia), characterized by surficial cover conditions comparable to those of the Mars 2020 Landing Site. The scoring system is the same used for the Mars 2020 Landing Site (Fig. 6). Cretaceous, Bayanzag (Mongolia). (B) Bioturbation ichnofossil (*Ophiomorpha*) from the uncovered bedrock unit figured in A. (C) Spreiten burrow from the uncovered bedrock unit figured in A. (D) Surficial unit (*K* = 2) partially covering a horizontal burrow. Miocene, Taza-Guercif Basin (Morocco).

**Selection criteria—** The identification of ichnological sites on Earth is based on the observation of the bedrock where eventual ichnofossils are found. Surficial cover, consisting of unconsolidated superficial deposits covering solid rock, hampers the observation of the bedrock, thus precluding the detection of eventual ichnofossils (Fig. 5). Surficial cover (regolith, dune systems), if present, precludes the observation of the Martian bedrock as well, thus preventing the observation of eventual ichnofossils. Consequently, the surficial cover is selected as a suitability factor for ichnosite location.

Recent erosional phenomena (*e.g.*, wind weathering) are also known to influence trace fossil preservation on Earth (Henderson, 2006). However, the impact of wind erosion is difficult to predict because it requires to quantify either the present or the past prevailing wind direction and intensity. This is further complicated by the fact that Mars differs from Earth in several weather-related parameters, *i.e.*, its greater distance from the Sun, its smaller size, its lack of liquid oceans and its thinner atmosphere, composed mainly of CO_2_ (Henderson, 2006; Read, Lewis & Mulholland, 2015). In addition, wind erosion can obliterate but also enhance the visibility of trace fossils *via* selective weathering processes. Areas that are highly exposed to winds may not necessarily less suitable than sheltered ones. For these reasons, there is a high risk of overinterpreting the effect of wind on ichnofossil suitability. Consequently, we did not include it in the predictive model.

**Proxies for spatial distribution —** The distribution of surficial cover across the Mars 2020 Landing Site can be deduced from the photogeologic map of Stack et al. (2020), which presents the distribution of surficial units. HiRISE imagery allows us to understand the degree to which each surficial unit covers the bedrock.

**Figure 6:**
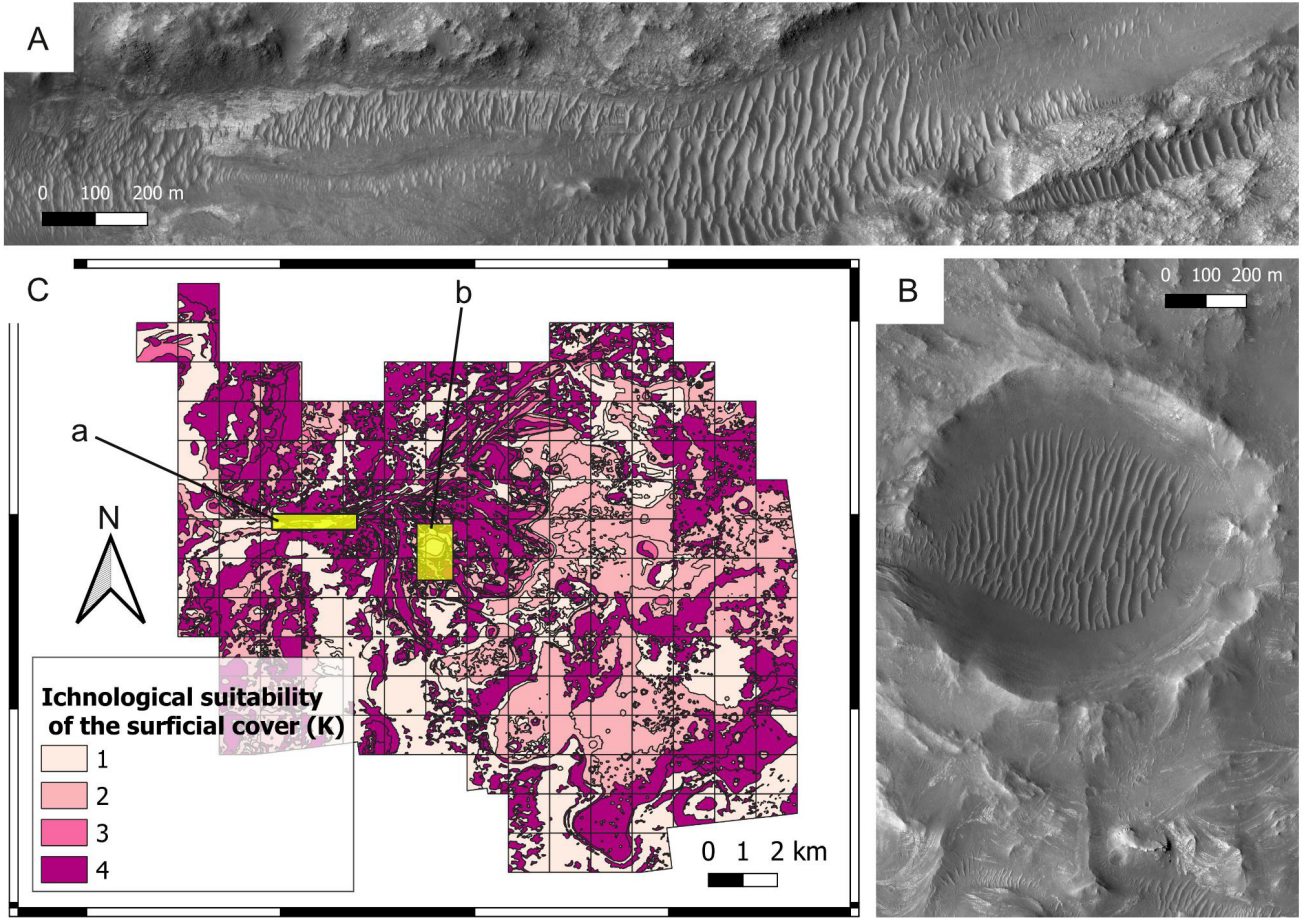
Ichnological suitability of the surficial cover (*K*) at the Mars 2020 Landing Site. (A) Surficial deposits (aeolian bedforms, large unit) covering the bedrock of the Neretva Vallis. A low suitability score (*K* = 1) is attributed to the surficial deposits, whereas optimal observation conditions are associated with the uncovered bedrock (margin fractured unit) at the edges of the valley. The location of the image is shown in C. HiRISE image. (B) Surficial cover within and around the Belva Crater. Observation of the crater floor is hampered by aeolian deposits (aeolian bedforms, large unit), which are assigned a low suitability score (*K* = 1). The crater rim is covered by the undifferentiated smooth unit. The location of the image is shown in C. HiRISE image. (C) Suitability map of the surficial cover conditions. The map shows the suitability of the surficial cover conditions for the observation of eventual ichnofossils. The insets labelled with a and b indicate the areas detailed in A and B, respectively.

**Suitability scoring —** Surficial units are attributed suitability scores ranging from 1 to 4, with 4 representing the most desirable cover conditions (*i.e.*, uncovered bedrock) and 1 the least desirable ones (*i.e.*, completely covered bedrock). Comparison with the Bayanzag ichnosite, which presents surficial units comparable with those found within the Jezero Crater, has been particularly informative for attributing suitability scores (Figs. 5A–5C). In fact, Cretaceous ichnofossils can be observed within the talus deposits of Bayanzag, consisting of fragmented bedrock accumulated at the base of the cliffs (Fig. 5A). Based on this observation, the talus deposits of the Jezero crater are assigned a relatively high score notwithstanding their nature of surficial cover. The aeolian and deflation units of Bayanzag also find immediate analogies with the aeolian (large and small) and undifferentiated smooth units of the Mars 2020 Landing Site. These surficial units significantly hamper the observation of ichnofossils, therefore a similar unsuitable condition is assumed for the areas of the Jezero crater covered by large aeolian bedforms (*e.g.*, Neretva Vallis, crater floor) (Fig. 6). These areas are, therefore, unsuitable for the detection of ichnofossils, if any. By contrast, large areas of the Western Delta are uncovered, allowing the observation of the Martian bedrock and of the eventual ichnofossils preserved within it.

### Energy regime

**Selection criteria —** Fluid flow is one of the most widespread transport and deposition processes in both subaerial and aqueous sedimentary environments. On Earth, the importance of fluid flow is exemplified by the pervasive action of currents, waves, and winds, among other processes, over the type and mobilization of the substrates. Geological evidence indicates persistent water flow on Early Mars (Malin & Edgett, 2003), as well as for ancient wind activity (Banham et al., 2018). Any flowing fluid possesses energy due to its motion, which is often referred as to hydrodynamic energy in the case of flowing water. The energy of a flowing fluid is one of the most common limiting factors in trace fossil distribution on Earth, influencing both the tracemaker behaviour and the preservation potential of ichnofossils (Buatois & Mángano, 2011). The energy regime is chosen as a selection criterion because it influences the suitability for ichnofossils independently from the planetary locale. In fact, substrate particles are increasingly removed from the bottom as the energy increases (Allen, 1992; Nichols, 2009; Duran Vinent et al., 2019). Particle removal is independent from the physical or biogenic nature of the sedimentary structures preserved within the substrate, *i.e.,* a burrow obeys to the same physical rules governing, for instance, the preservation of ripples or mudcracks. Consequently, high-energy conditions tend to obliterate pre-existing fabrics and sedimentary structures regardless of their biogenic or abiogenic nature. This phenomenon depends solely on sediment and flow dynamics, holding on Earth and on Mars.

**Proxies for spatial distribution —** The spatial variability of the past energy conditions at the Mars 2020 Landing site is here deduced from sedimentary architecture, grain size and landforms. Deltas are formed by deceleration of the river outflow into a basin with a standing body (Postma, 1990; Postma, 2003), therefore the distance from the mouths of the palaeorivers is used as a reliable indicator of hydrodynamic energy in the Jezero palaeolake. Grain size is also used as a proxy for hydrodynamic energy because transport of sediments occurs when the currents are high enough for the bed shear stress to exceed the threshold of motion, which depends upon the median sediment grain size (Ward et al., 2020). An additional hydrodynamic proxy is the geomorphic evidence of channels, which are recognized as areas of high-velocity flow in fluvio-deltaic systems (Shaw, Mohrig & Wagner, 2016). Since hydrodynamic energy usually decreases with increasing water depth, the current elevation of the landing site is also taken as a proxy for hydrodynamic conditions.

**Suitability scoring —** The bedrock units of the landing site are attributed scores from 1 to 4, with 4 representing the best (lowest-energy) hydrodynamic conditions for ichnosite location.

**Figure 7:**
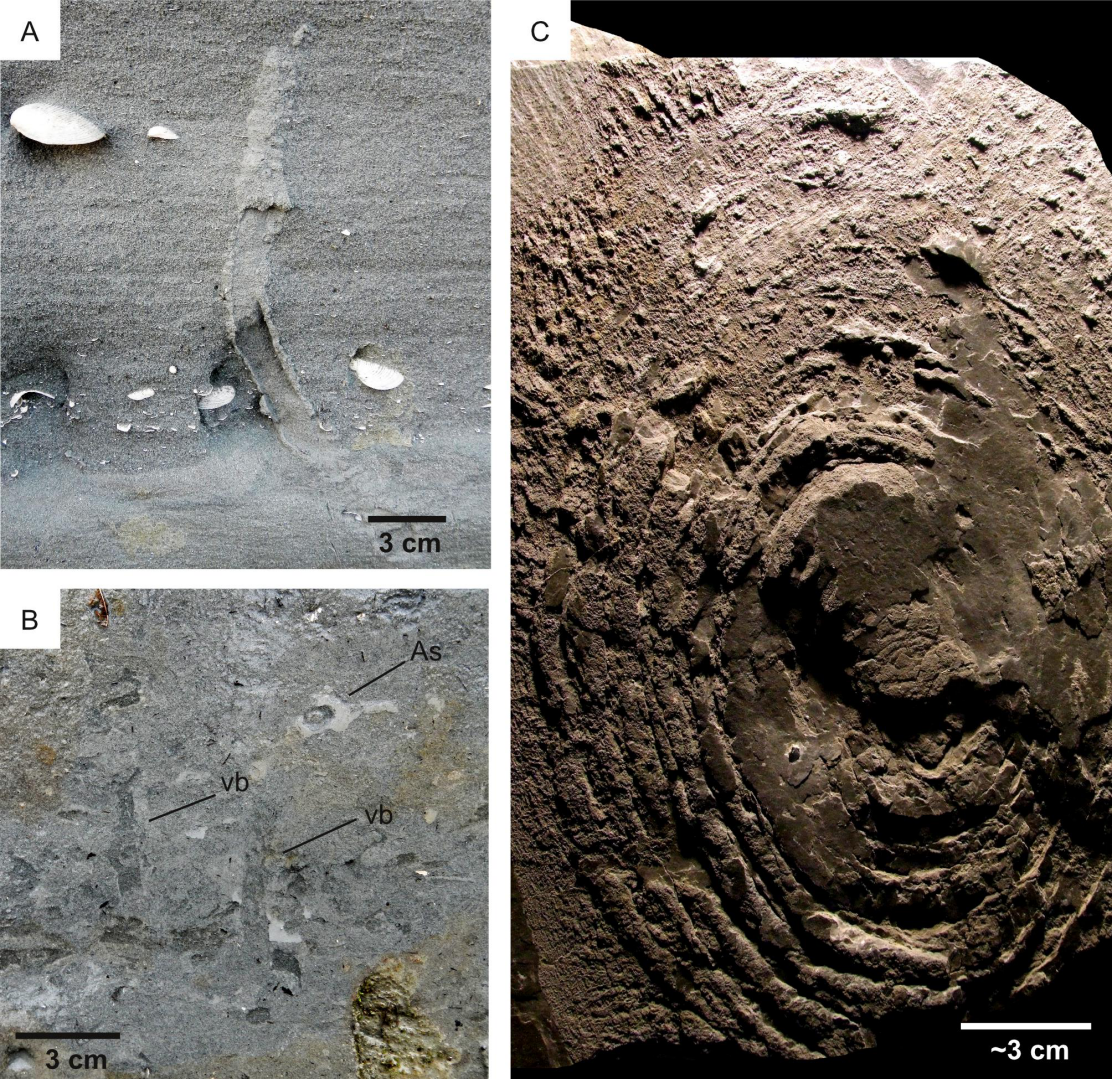
Ichnological suitability of the energy regime (*E*). (A–B) Ichnofabrics along a hydrodynamic gradient from high- (A) to low-energy (B) deltaic environments of the Arda River (Pleistocene, Italy). High-energy deposits (*E* = 4) are characterized by *Ophiomorpha* and low bioturbation intensity, whereas low-energy deposits (*E* = 1) are almost completely bioturbated. Only few vertical burrows (vb) and a horizontal one (*Asterosoma:* As) are visible. (C) Microbial-induced sedimentary structure from Middle Ordovician low-energy (*E* = 1) settings. Canelas, Portugal.

Low scores are attributed to high-energy settings because most surface or near-surface bioturbation traces are removed by erosion in high-energy environments, whereas the preservation potential increases with decreasing energy (Hallam, 1975; Curran, 1994; Bromley, 1996; Buatois & Mángano, 2011). This phenomenon is explained by the sedimentological nature of bioturbation structures, which are at one with the substrate before and after diagenesis (Hallam, 1975). The low ichnological suitability of high-energy settings is supported by observations at the Arda reference ichnosite, which encompasses a gradient from high-energy (fluvial, shoreface) to low-energy (offshore) fluvial-influenced settings (Crippa et al., 2018). High-energy deposits of the Arda section, Italy (Fig. 7A) display a lower bioturbation intensity than low-energy ones (Fig. 7B). Also, these high-energy deposits show how tracemakers need to invest in extra-efforts to cope with the shifting substrates associated to high energy conditions, *i.e.,* the producers of *Ophiomorpha* reinforced burrows with pellets (Fig. 7A).

**Figure 8:**
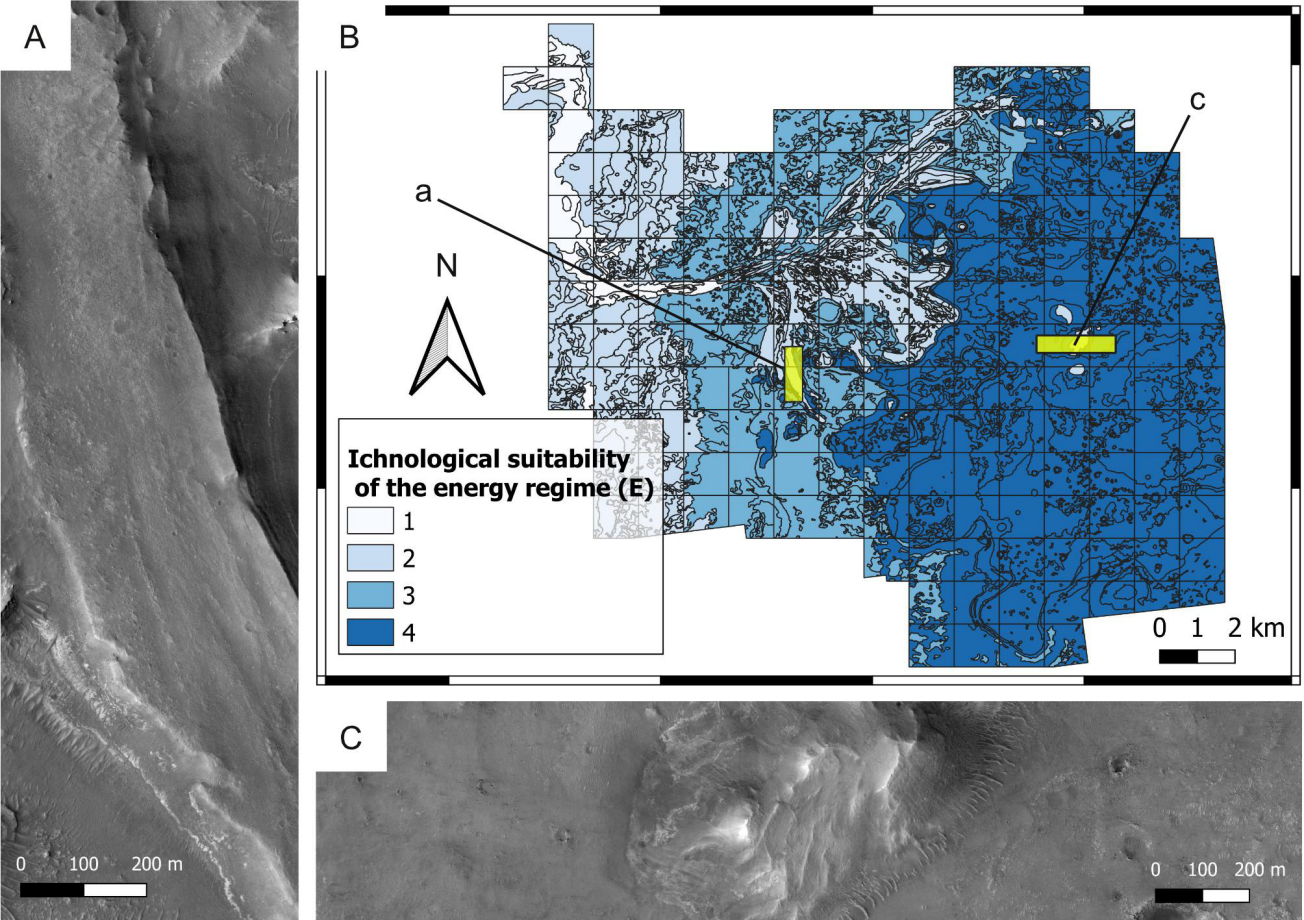
Ichnological suitability of the energy regime (*E*) at the Mars 2020 Landing Site. (A) High-energy, low-suitability (*E* = 2) channel deposit preserved as an inverted relief landform. The top of the ridge consists of the delta blocky unit, which is overlying the delta thinly layered unit. HiRISE image. (B) Suitability map of the kinetic energy conditions. The insets labelled with a and c indicate the areas detailed in A and C, respectively. (C) Distal area dominated by low-hydrodynamics, high-suitability conditions. The flat area consists of the crater floor fractured rough unit (*E* = 4), whereas the hill is a delta remnant consisting of the delta thinly layered (*E* = 4) and the delta blocky unit (*E* = 2). HiRISE image.

**Figure 9:**
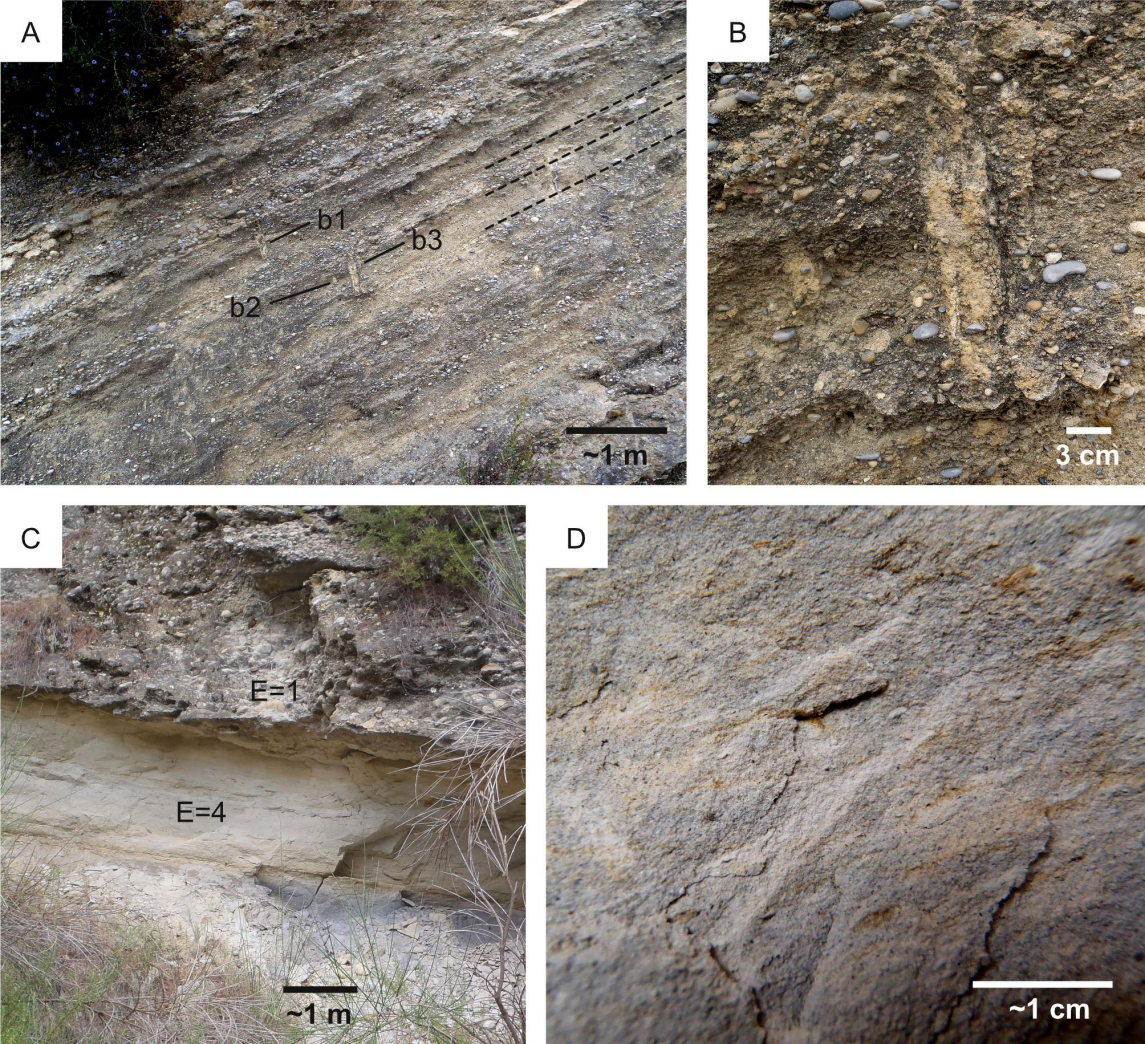
High-energy vs low-energy conditions in the Ventimiglia palaeodelta (Pliocene). (A) Alternation of conglomerates and sandstone deposited by a highly sustained flow (*E* = 1) along the delta slope. Few vertical burrows (b1-3) intersect the foresets, some of which are highlighted by a dashed line. (B) Detail of the burrows (b2-3) figured in A. (C) High-energy conglomerate unit overlying a low-energy marly unit. High-energy conditions are poorly suitable for ichnofossils (*E* = 1), whereas low-energy ones favour bioturbation (*E* = 4). This is supported by the absence of ichnofossils in the conglomerate unit and the bioturbated nature of the marly one. (D) Detail of the marly unit in C showing the branched burrow *Chondrites*.

Based on these observations, high suitability scores for bioturbation are attributed to the relatively quiet, distal areas of the delta, characterized the delta thinly layered and delta layered rough unit. By contrast, lower scores are attributed to the proximal areas of the Jezero delta, often characterized by channelized deposits consisting of the coarse-grained delta blocky unit (Fig. 8). This scoring is supported by the observations made at the Ventimiglia palaeodelta (Pliocene, Italy). Here, high-energy deltaic deposits are unbioturbated or sparsely bioturbated (Figs. 9A–9C), whereas higher bioturbation intensities are associated with lower energy ones (Fig. 9D). Similarly, the high-energy regime of the Neretva Valley, consisting of an incised fluvial channel, is interpreted as being particularly unsuitable for ichnofossil preservation.

By removing substrate particles, high-energy conditions negatively influence the preservation of bioerosion and biostratification structures as well. However, high-energy conditions have a lesser influence on the preservation of bioerosion and biostratification structures because these are related to more cohesive, erosion-resistant substrates. This aspect is addressed in the data aggregation stage (step 5) of the workflow, *i.e.,* a small weight is attributed to the energy regime when calculating the overall bioerosion and biostratification suitability. A higher weight is attributed to the energy regime for bioturbation suitability.

More in detail, this choice is justified by the fact that bioerosion ichnofossils necessarily develop in hardgrounds, which are less prone to erosion than softgrounds by their nature of lithic substrates. For this reason, the energy regime is not regarded among the major factors influencing the suitability for (eventual) bioerosion ichnofossils in the Mars 2020 Landing Site. In parallel to bioerosion ichnofossils, biostratification structures tend to withstand high-energy conditions better than bioturbation ichnofossils. This is explained by the fact that biostratification tend to stabilize depositional surfaces and shelter the sediment against erosion (Noffke et al., 2001). As a result, biostratification structures are common in a wide hydrodynamic range. Modern subtidal Bahamian stromatolites are positively associated with strong tidal currents because these are unfavorable for competitors such as metazoans and macroalgae (Noffke & Awramik, 2013). Exclusion of competitors is also the reason explaining why stromatolites are common in hypersaline lagoons, exceedingly warm waters and macrotidal settings (Noffke & Awramik, 2013; Suosaari, Reid & Andres, 2019). Some MISS are associated to bland erosional regimes, *e.g.*, erosional pockets are produced where pieces of microbial mat are removed by erosion, leaving an irregularly shaped mat border surrounding a depression through which the underlying sediment is exposed (Bose & Chafetz, 2009). Despite their resilience to erosion, biostratification structures tend to be more delicate than bioerosion ichnofossils because of their nature of sedimentary structures. Low-energy conditions favor the preservation of biostratification structures, as exemplified by the concentric biostratification structures of the Canelas reference ichnosite (Ordovician, Portugal). Here, taphonomy of body fossils indicate extremely low-energy conditions, which aided the excellent preservation of microbial-related biostratification structures (Fig. 7C) (Neto de Carvalho et al., 2016). Another example is provided by the Miocene palaeocoast of the Rio Negro Formation (Argentina), which was differentiated into high-energy erosional domains and protected areas where MISS became preserved (Carmona et al., 2012). Based on these observations, the high-energy deposits of the Jezero crater are here interpreted as less suitable for biostratification than low-energy ones. This parallels the impact of the energy regime on the suitability for bioturbation structures. However, because of the mentioned substrate-stabilizing effect, a relatively low weight is assigned to the suitability score of the energy regime for biostratification. Weight is used in overlay analysis, during which a weighted sum is computed across multiple layers to account the fact that some suitability factors are more influential than others in biostratification structure distribution.

**Figure 10:**
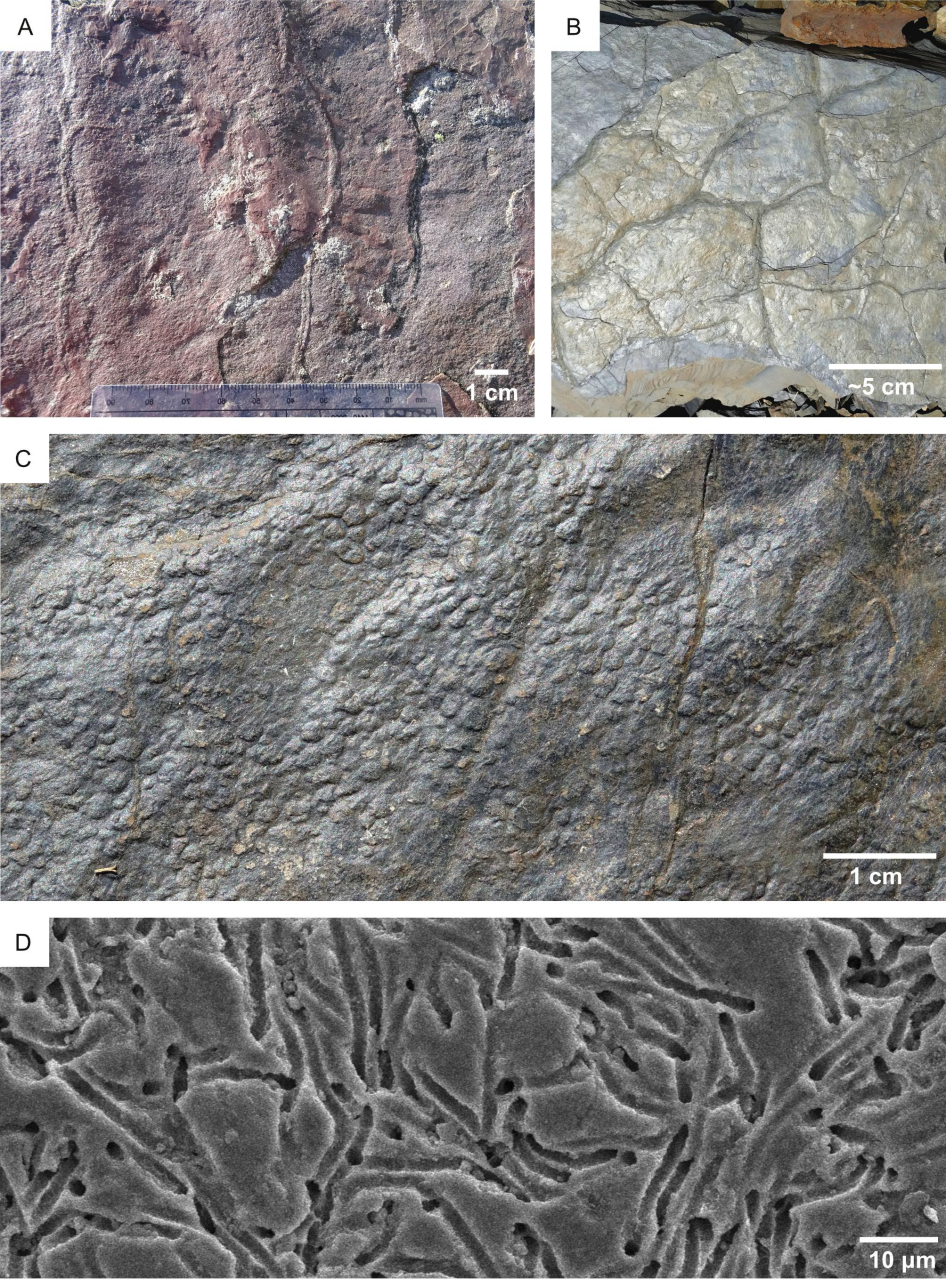
Ichnofossils in fluvio-lacustrine substrates on Earth. (A) High-suitability conditions for bioturbation (*L* = 4) are suggested by ripples, which require non-cohesive substrates for their formation. Note locomotion ichnofossils (burrows). The photo refers to the Collio Basin (Permian, Italy), which was characterized by ephemeral shallow lake conditions under a semiarid to arid climate, interested by density underflows at fan toes. (B) High-suitability conditions for bioturbation (*L* = 4) are suggested by mudcracks, which are formed contraction of a non-cohesive substrate. Mudcracked surface indicating softground conditions (*L* = 4). Collio Basin (Permian, Italy). (C) Elephant skin, a microbial mat growth structure. Detail of the mudcracked surface in B. (D) Freshwater hardground (*H* = 4) bored by cyanobacteria of the family Pseudanabaenaceae. The hardground consists of a modern gastropod from the La Brava lake (Argentina). Photo from Tietze & Esquius (2018).

**Figure 11:**
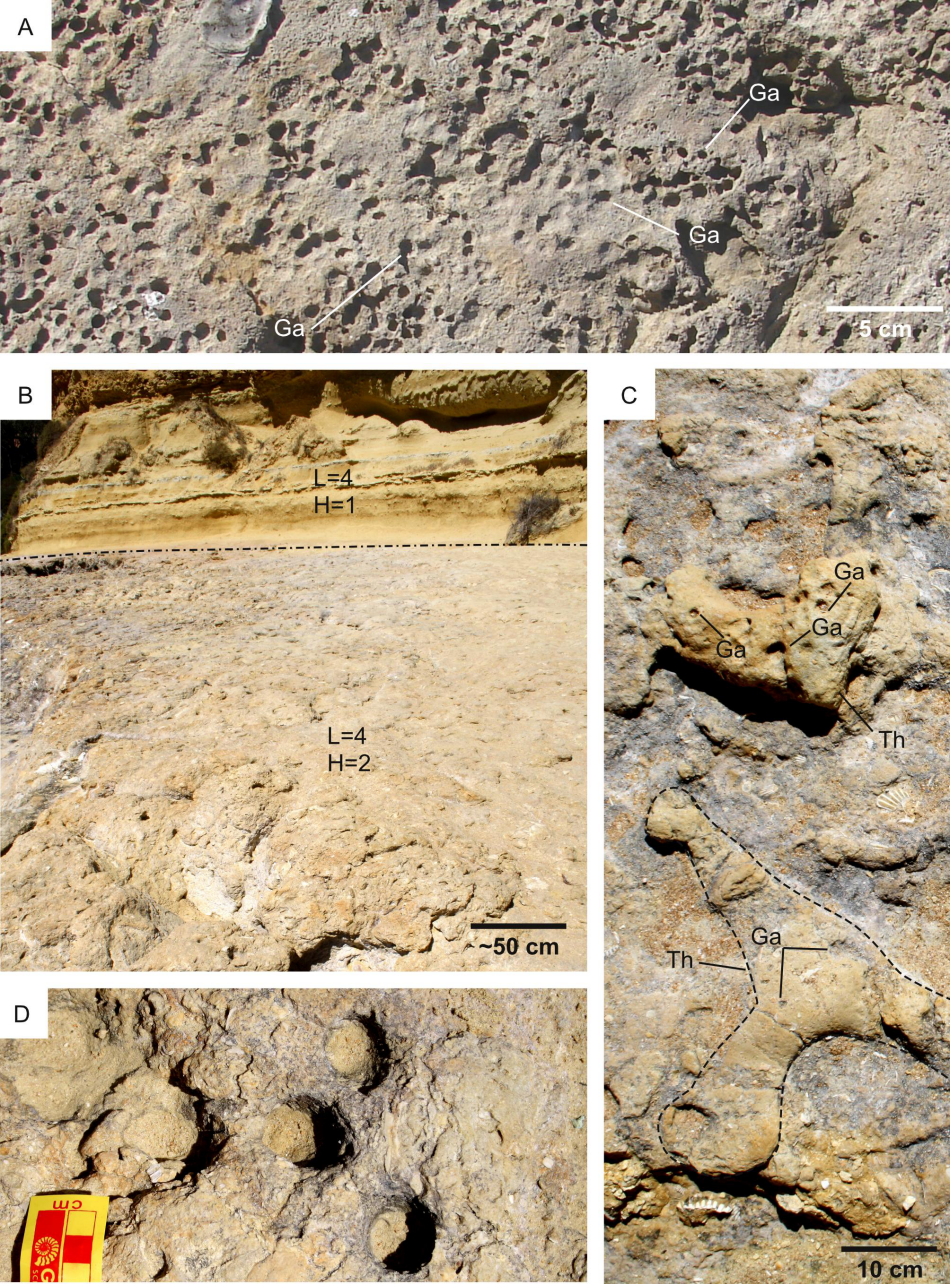
Substrate suitability for bioerosion (*H*) and bioturbation (*L*). (A) Cretaceous (Albian) hardground displaying high substrate suitability for bioerosion (*H* = 4). The hardground bears abundant *Gastrochaenolites* borings (Ga). Top view; Parede (Portugal). (B) Unconformity surface suitable for both bioturbation (pre-lithification suite) and bioerosion (post-lithification) ichnofossils (*L* = 4, *H* = 2). The unconformity developed within a Serravallian bioturbated softground that successively undergo lithification and bioerosion. The dash-dot line separates the unconformity surface from the softground (*L* = 4, *H* = 1) deposited during the Tortonian. Oura (Portugal). (C) Bioeroded bioturbation ichnofossils from the Oura unconformity surface (Miocene, Portugal). The bioerosion ichnofossils are *Gastrochaenolites* (Ga), the bioturbation ichnofossils consist of *Thalassinoides* (Th), one specimen of which is highlighted by a dashed line. The figure is a detail of B. (D) *Gastrochaenolites* from the Oura unconformity, shown in B and C.

### Substrate cohesiveness

**Selection criteria —** Substrate cohesiveness is selected as a suitability factor because the mechanical properties of the substrate constrain the ichnofossil type that can (eventually) be produced. On Earth, the mechanisms of moving through solid substrata depend on the mechanical properties of the substrate (Dorgan, 2015). Organisms move through loose substrates by displacing sediment grains (bioturbation; Fig. 10A); they move by creating an opening in hard substrata by mechanical or chemical means (bioerosion; Fig. 11A) (Bromley, 1996; Dorgan, 2015). As a result, the cohesiveness of a given substrate constrains the type of ichnofossils that can be produced within it, *i.e.,* bioturbation structures can be produced only in unconsolidated substrates because hardgrounds do not provide grains to displace. This relationship between substrate cohesiveness and ichnofossil type holds not only for Earth but also for Mars because it derives solely from the mechanical properties of the substrate, being independent of the planetary locale on which the substrate is found. This is a fact proved by the traces left by the Curiosity rover, producing ‘bioturbational’ trails on Martian softgrounds and ‘bioerosional’ drill holes into hardgrounds (Baucon et al., 2017). Consequently, the presence of softgrounds is here regarded as a suitability factor for bioturbation ichnofossils, whereas the presence of hardgrounds is a suitability factor for bioerosion. For this reason, the impact of the substrate on ichnofossil suitability is accounted by two different variables, *L* (substrate suitability for bioturbation) and *H* (substrate suitability for bioerosion; Table 1). When calculating the overall suitability for bioturbation (*A*; Table 1), *H* is ignored, and *L* contributes to the weighted sum. Conversely, *L* does not contribute to the weighted sum for bioerosion suitability (*B*; Table 1).

Whereas substrate cohesiveness constrains the development of bioerosion and bioturbation structures, it exerts less influence on biostratification. This is counterintuitive because biostratification acts on loose particles, which however can be found not only within softground substrates but also in the water column. For instance, the most commonly cited pathway for Phanerozoic marine stromatolites is the trapping and binding model, according to which successive generations of microbial filaments (or extracellular polymeric substances) trap grains settling from the water column and bind the sediments *via* precipitated cements (Shapiro, 2007).

Substrate type does not necessarily limit movements of biostratification-forming organisms, *e.g.*, cyanobacteria on Earth move through the sediment that blanketed them by jet gliding upwards in the secreted exopolysaccharides (Hoiczyk, 2000; Foster et al., 2009). The weak influence of substrate type on biostratification is supported by the fact that fossil and modern stromatolites are reported from both softgrounds and hardgrounds (Reid et al., 1995; Stefano et al., 2002). It should be however noted that MISS are restricted to softgrounds by their nature of sedimentary structures. This is exemplified by the lacustrine MISS of the Collio Formation, Italy, which are dissected by mudcracks, being therefore related to softgrounds (Figs. 10B–10C).

**Proxies for spatial distribution —** The cohesiveness of the ancient substrate is deduced from the sedimentological characteristics of the bedrock units. Specifically, deltaic deposits are interpreted as proxies for softgrounds based on the fact that delta formation requires unconsolidated sediments. Conversely, deposits that predate the Jezero impact are interpreted as hardgrounds. Carbonate-rich units were deposited as softgrounds, but the terrestrial record (Knaust, Curran & Dronov, 2012) show that they can undergo early cementation. For this reason, deposits rich in carbonate are taken as a proxy for both softground and hardground conditions.

**Figure 12:**
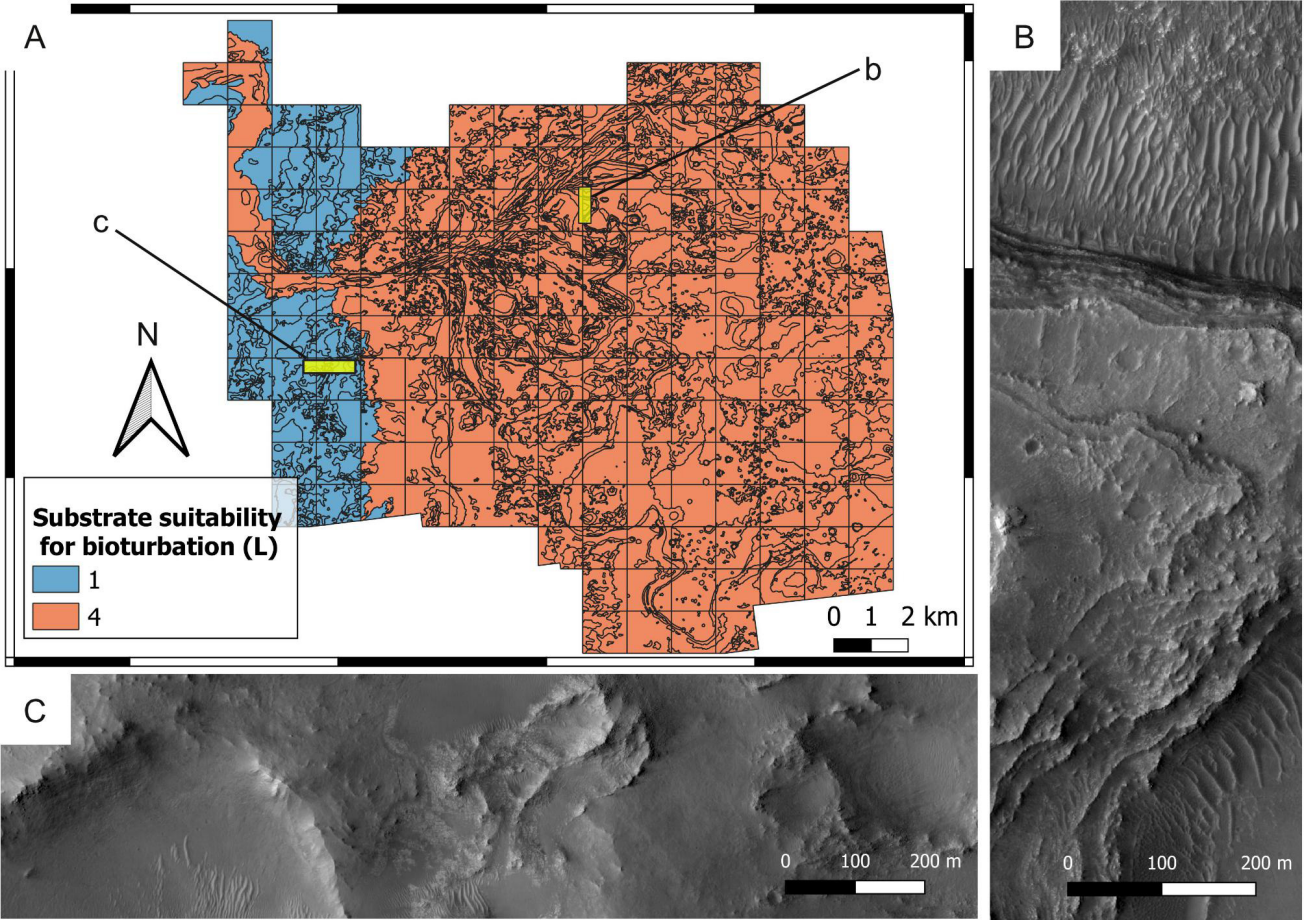
Suitability map of the substrate type for bioturbation ichnofossils. (A) Suitability map of the substrate type for bioturbation ichnofossils. The insets labelled with b and c indicate the areas detailed in B and C, respectively. (B) Layered deposits that deposited as softgrounds, being suitable (*L* = 4) for bioturbation. They consist of the delta thinly layered unit. HiRISE image. (C) Substrate that was lithified at the time of the Jezero palaeolake, being unsuitable (*L* = 1) for bioturbation. The imaged area comprises the crater rim blocky unit. HiRISE image.

**Figure 13:**
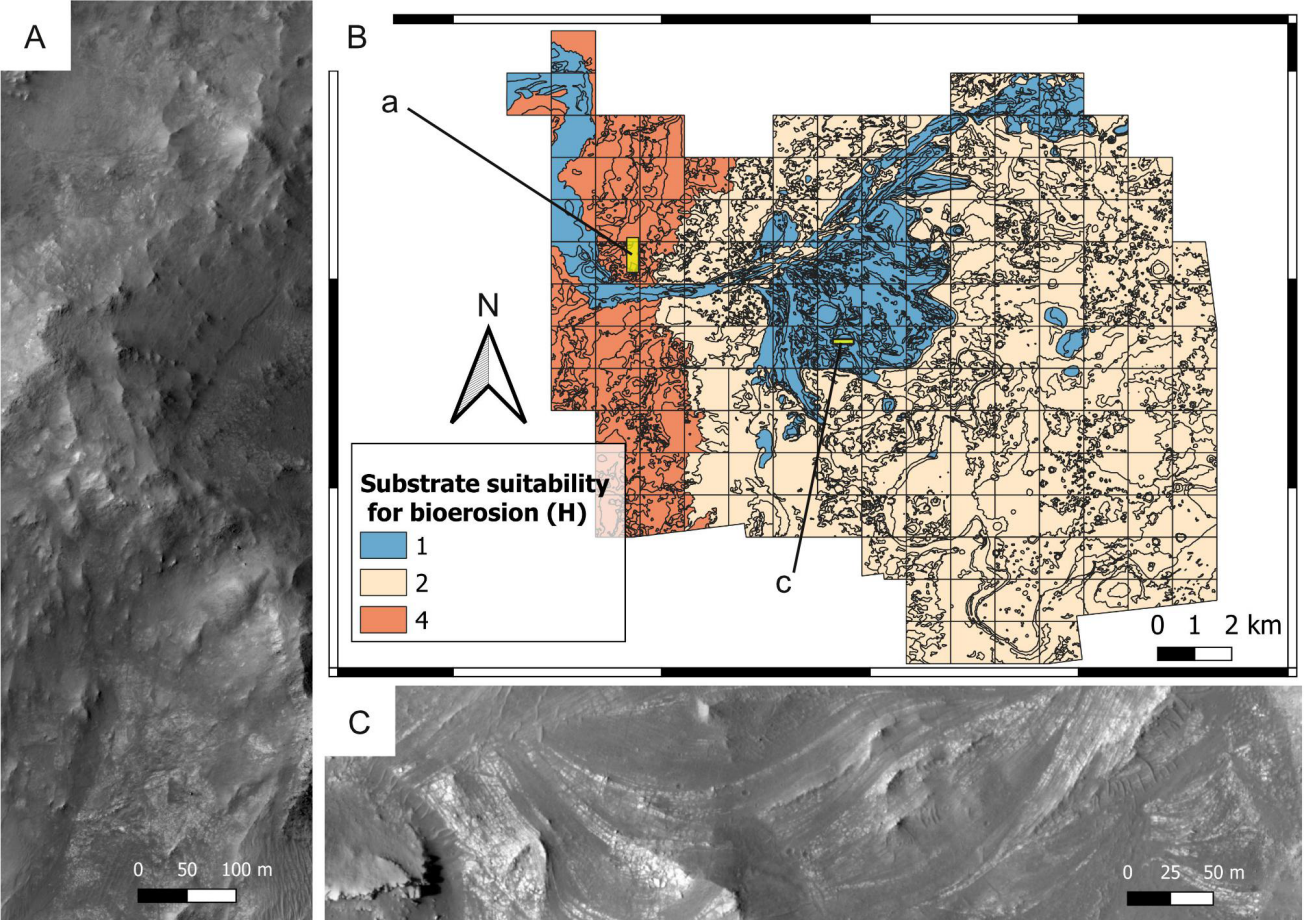
Suitability of the past substrate type (*H*) for bioerosion. Mars 2020 Landing Site. (A) Outcrop of the crater rim blocky unit, which was plausibly lithified at the time of the Jezero palaeolake, being therefore suitable (*H* = 4) for bioerosion. HiRISE image. (B) Suitability map of the substrate type for bioerosion ichnofossils. The insets labelled with a and c indicate the areas detailed in A and C, respectively. (C) Deltaic unit deposited as a softground, being therefore unsuitable (*H* = 1) for bioerosion. The outcrop exhibits deposits of the delta truncated curvilinear layered unit. HiRISE image.

**Suitability scoring —** Bedrock units have been attributed bioturbation suitability scores ranging from 1 to 4, with 4 representing the most desirable substrate conditions (*i.e.,* loose substrates) and 1 the most unsuitable conditions (*i.e.,* hardgrounds) (Fig. 12). A specular scoring system is used for bioerosion, *i.e.,* 4 represents the most suitable conditions (hardgrounds) and 1 the most unsuitable ones (loose substrates) (Fig. 13).

Deltaic units are assigned high bioturbation scores because they necessarily derive from the deposition of unconsolidated sediments, allowing benthic organisms, if any, to displace sediment grains (Fig. 10B). By contrast, the crater rim units are attributed low suitability scores for bioturbation (Fig. 12) because they are part of the basement sequence that predates the formation of Jezero crater (Stack et al., 2020). Consequently, they plausibly represented hardgrounds at the time of the Jezero palaeolake. For the same reason, the crater rim is attributed a high suitability score for bioerosion (Fig. 13). Hard substrates were also provided by the crater rim breccia, which has been interpreted as an impact breccia formed during the Jezero impact event or the Isidis event (Stack et al., 2020).

The above suitability scores are based on the fact that bioturbation and bioerosion require opposite substrate conditions (*e.g.*, hardgrounds cannot be bioturbated). In most cases, sites with a high bioturbation score (*L* = 4) are attributed a low bioerosion score (*H* = 1), and vice-versa. However, the ichnofossil record of the Earth shows that, under specific environmental conditions, the same site can be suitable for both bioturbation and bioerosion ichnofossils. For instance, the unconformity surface of the Oura reference ichnosite (Portugal) displays crustacean bioturbation ichnofossils (*Thalassinoides*) intersected by bivalve bioerosion ichnofossils (*Gastrochaenolites)* (Figs. 11B–11D). The unconformity developed within a Miocene (Serravallian) bioturbated softground that successively lithified and formed a rocky shoreline, which was subsequently bioeroded (Cachão et al., 2009). A similar scenario is possible for the crater floor units of the Mars 2020 Landing Site, which may be cross-cut by an unconformity surface (Stack et al., 2020). Unconformities tend to become colonized by substrate-controlled trace fossil suites when exposed to aqueous conditions (Pemberton et al., 2001; MacEachern et al., 2007; Buatois & Mángano, 2009; Richiano et al., 2019). Similarly, the richness in carbonate of the margin fractured unit (Ehlmann et al., 2008b; Goudge et al., 2015; Horgan et al., 2020) may have favored fast diagenesis, producing a softground-to-hardground transition.

High suitability scores for bioturbation are attributed to the crater floor deposits based on their possible nature of fluvio-lacustrine softgrounds. This interpretation is supported by the contact of crater floor fractured 2 unit with the deltaic units, as well as the textural similarities between crater floor fractured unit 1 and 2 (Stack et al., 2020). However, it should be highlighted that other plausible interpretations are available, *e.g.*, lava flows, magmatic intrusions, impact condensates, tephra deposits, aeolian, airfall and fluvial deposits (Hoefen, 2003; Ody et al., 2013; Bramble, Mustard & Salvatore, 2017; Palumbo & Head, 2018; Rogers et al., 2018; Kremer, Mustard & Bramble, 2019; Mandon et al., 2020; Stack et al., 2020).

### Sedimentation rate

**Selection criteria —** Sedimentation rate has long been recognized as among the major influences on the intensity of bioturbation, *i.e.,* the degree to which the original fabric of the substrate has been modified by organisms (Bromley, 1996; Taylor, Goldring & Gowland, 2003; Buatois & Mángano, 2011). Data from Earth show that very low bioturbation intensities commonly correlate to elevated rates of sedimentation and massive bedding, while high bioturbation intensities are usually associated with slow sedimentation and heterolithic deposition (Gingras, MacEachern & Dashtgard, 2011). Low/null sedimentation also enables the colonization of hardgrounds by boring organisms (Łaska, Rodríguez-Tovar & Uchman, 2021). The relationship between sedimentation rate and the degree of biological reworking is explained in terms of availability of time, *i.e.,* the degree to which a substrate is biologically reworked depends on the amount of time available for biogenic activity per unit accumulation of sediment (Howard, 1975). In other words, slow sedimentation rates provide organisms with a longer amount of time for reworking (bioturbating or bioeroding) the substrate. This phenomenon is not dependent upon the planetary locale, therefore, sedimentation rate is here selected as a suitability factor for the Mars 2020 Landing Site.

**Proxies for spatial distribution —** On Earth, the sedimentation rate can be estimated by considering the thickness of a given sedimentary unit and the amount of time in which the unit deposited. These variables cannot be precisely estimated for the geological units of the Mars 2020 Landing Site; however, the identification of the major sources of sediment allows a qualitative estimate of the spatial variability of the sedimentation rate across the study area. It is not possible to provide absolute values for the sedimentation rate, but it is possible to assess the relative values based on the different architectural elements of the Jezero delta. In this regard, only two fluvial inlets entered the Jezero lake, bringing sediments from a mineralogically-diverse area into the lake (Schon, Head & Fassett, 2012). Consequently, the areas of maximum sedimentation coincided with the deltaic areas adjacent to the river mouths; conversely, the areas with the lowest sedimentation rates were located far away from the river mouths. This interpretation is supported by investigation of terrestrial deltas. These are not exact analogues of the Jezero delta but necessarily share similar sedimentary dynamics due to the rapid deceleration of water flow at the river mouth. For example, in the Fraser River delta, Canada, the maximum sedimentation (∼13 cm yr^−1^) occurs in the vicinity of the river mouth (Hart, Hamilton & Barrie, 1998; Ayranci & Dashtgard, 2013). Anyway, in deltaic systems where density currents can occur regularly, a significant proportion of riverine sediment input may be transferred to the distal part of the systems leading to important distal sediment accumulation zones (distal delta lobe). A sediment budget was calculated for the Rhone River delta system, in eastern Lake Geneva, Switzerland (Silva et al., 2019). Mean sedimentation rates in these areas vary from 0.0737 m year^−1^ (delta front) to 0.0246 m year^−1^ (distal delta lobe). The remaining area, lake basin background deposition, show sedimentation rates one order of magnitude smaller.

**Figure 14:**
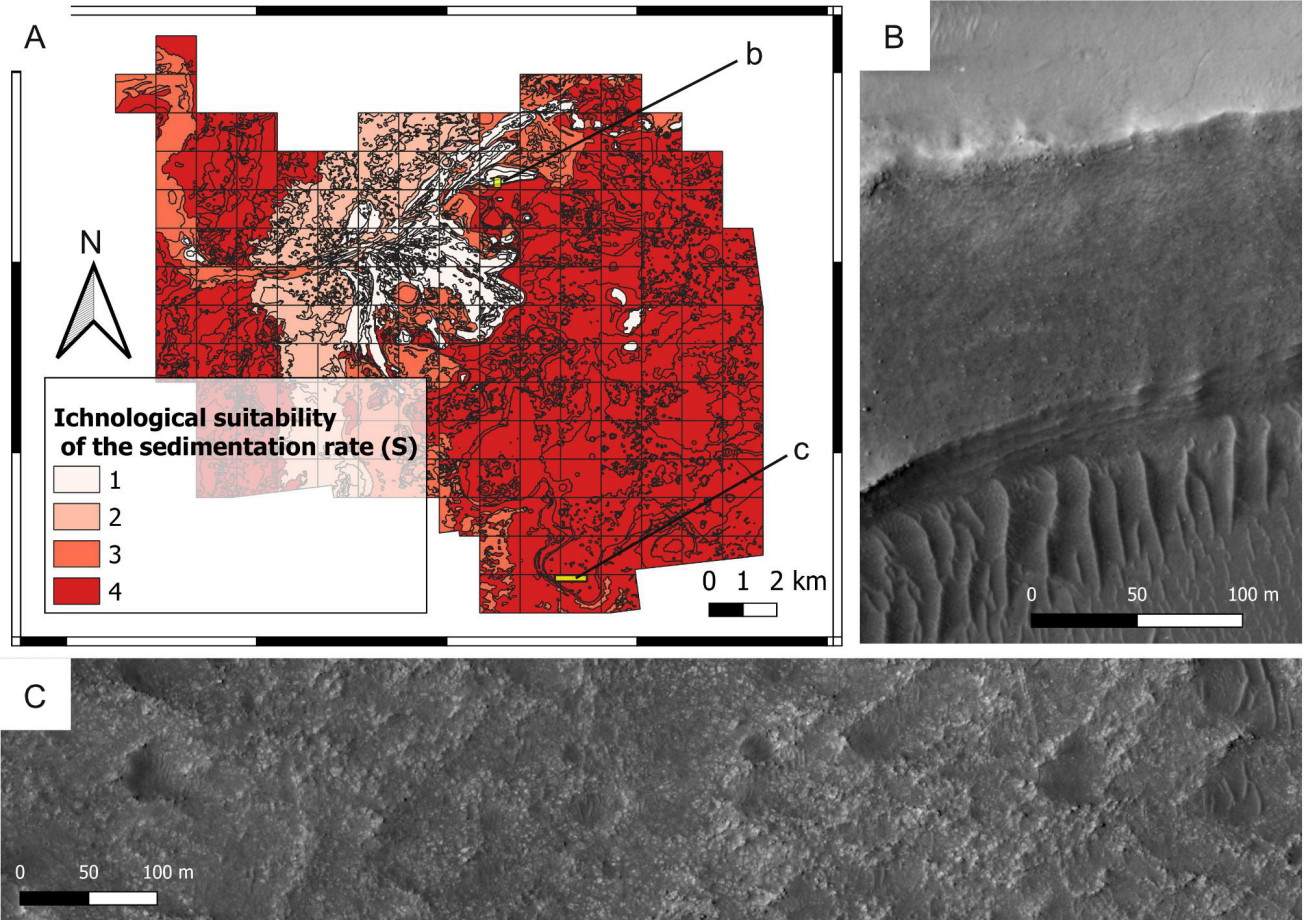
Suitability of the sedimentation rate conditions (*S*) in the Jezero crater. (A) Suitability map of the sedimentation rate for ichnofossils. The insets labelled with b and c indicate the areas detailed in B and C, respectively. (B) High-sedimentation deposit overlying a well-bedded, lower-sedimentation one. The high-sedimentation deposit consists of the delta blocky unit, the low-sedimentation one is the delta thinly layered unit. HiRISE image. (C) Distal sector of the study area, that plausibly was subject to low sedimentation rates. The outcrop consists of the crater floor fractured rough unit. HiRISE image.

**Figure 15:**
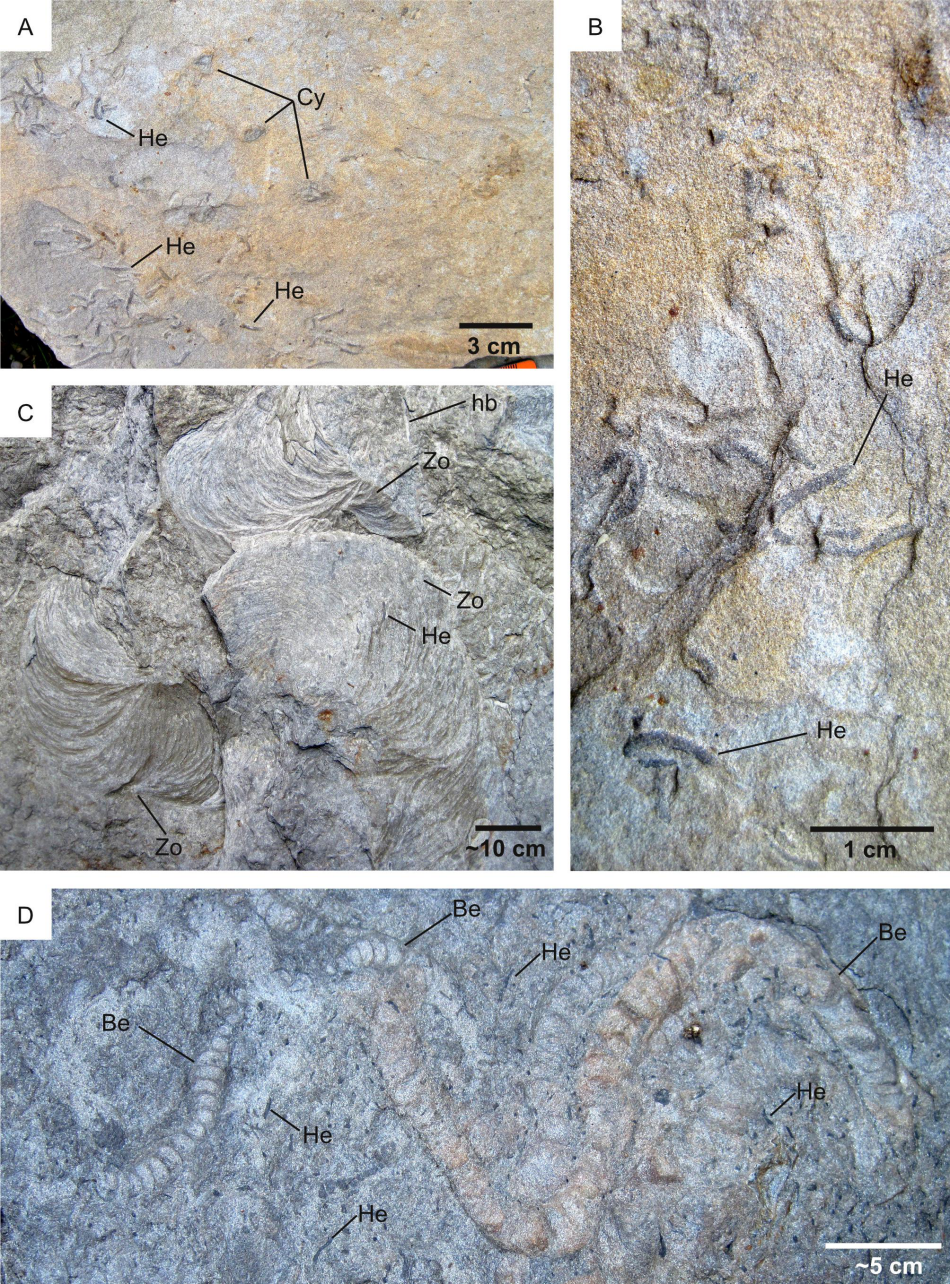
Ichnofossils along a sedimentation rate gradient. Pramollo Basin (Carboniferous, Italy-Austria). Bedding plane views. (A) Delta-front unit deposited in a high-sedimentation setting, thus presenting a low suitability for ichnofossils (*S* = 1). The ichnofossils *Helminthoidichnites* (He) and *Cylindrichnus* (Cy) occupy a small area of the bedding plane. (B) Detail of A showing *Helminthoidichnites* (He). (C) Offshore deposits characterized by low sedimentation rate and, therefore, a high ichnological suitability (*S* = 4). The bedding plane is intensely bioturbated by horizontal burrows (hb) and *Zoophycos* (Zo). (D) Offshore deposits characterized by low high (*S* = 4) bioturbation suitability. The bedding plane is completely covered by ichnofossils, among which *Helminthoidichnites* (He) and *Beaconites* (Be).

**Figure 16:**
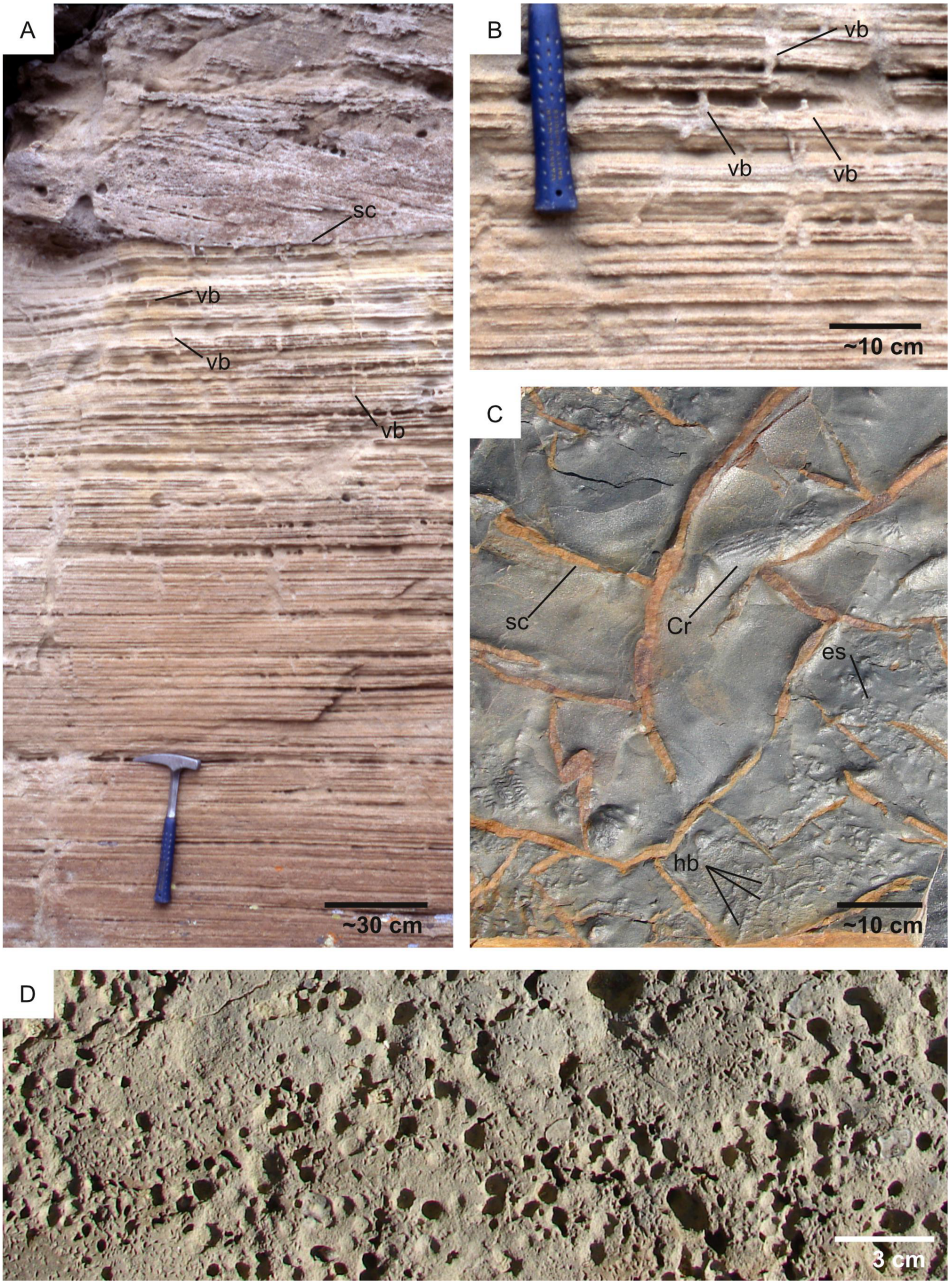
High- *vs* low-sedimentation rate. (A) Lacustrine delta deposits displaying a low-diversity association dominated by vertical burrows (vb). The excellent preservation of primary sedimentary structures indicate that sedimentation rate exceeded bioturbation rate. A scour surface (sc) is also observed in the upper part of the image. Little Muddy Creek area (Eocene, USA). Image from Zonneveld, Bartels & Clyde (2003). (B) Detail of the outcrop figured in A showing vertical burrows (vb). (C) Marine prodelta deposits with the burrow *Cruziana* (Cr), numerous horizontal burrows (hb) and MISS (elephant-skin texture: es). Shrinkage cracks (sc) are also observed. Penha Garcia (Ordovician, Portugal). (D) Intensely bioeroded hardground, having required low to null sedimentation rate for its formation. Parede (Cretaceous, Portugal).

**Suitability scoring —** The geological units of the study area have been attributed scores ranging from 1 to 4, with 4 representing the most desirable conditions (*i.e.,* low sedimentation rate) for bioturbation and bioerosion ichnofossils (Fig. 14). High scores are attributed, for instance, to the distal areas of the Jezero delta where sedimentation rate was plausibly low, providing eventual organisms with longer amounts of time for bioturbating the substrate. By contrast, the areas in the vicinity of the palaeoriver mouth are attributed low suitability scores. This suitability scoring is motivated not only by ichnological theory (Bromley, 1996; Taylor, Goldring & Gowland, 2003; Buatois & Mángano, 2011; Gingras, MacEachern & Dashtgard, 2011; Tonkin, 2012), but also by empirical observations at the Pramollo Basin (Carboniferous-Permian; Italy-Austria) (Baucon & Neto de Carvalho, 2008; Baucon et al., 2015b). Here, delta front deposits present lower bioturbation intensities than the units deposited in more distal locations of the same basin (Fig. 15). The same phenomenon is reported from lake settings, *i.e.,* sedimentation rate tends to exceed bioturbation rate in the more proximal sectors of lacustrine deltas (Figs. 16A, 16B) (Zonneveld, Bartels & Clyde, 2003; Buatois & Mángano, 2011). Conversely, higher bioturbation and bioerosion rates are normally associated with lower sedimentation rates (Figs. 16C, 16D).

### Water level

**Selection criteria —** On Earth, the availability of water is a fundamental control factor on ichnofossil formation (Hasiotis & Honey, 2000; Buatois & Mángano, 2011). This mirrors the astrobiological principle by which water is an essential compound for the existence of life as we know it (Mottl et al., 2007; Jones & Lineweaver, 2010). The presence of water is considered so important for life that the astrobiological exploration of Mars has been guided by the search for water (Hubbard, Naderi & Garvin, 2002; Grotzinger, 2009). In the Jezero Crater there are clear proxies for the past presence of liquid water (Salese et al., 2020), which therefore plausibly represented an abundant resource for organisms, if any. For these reasons, the presence of water has been selected as a suitability factor for the Mars 2020 predictive model, although it should be noted that extraterrestrial life without water can be conceived as well (Mottl et al., 2007).

**Proxies for spatial distribution —** The presence of water within the study area is deduced from sedimentological and geomorphological observations. In this regard, fluvio-deltaic landforms are clear indicators of the presence of water in a liquid state, for which reason there is little doubt about the aqueous environment of the Jezero deltas (Salese et al., 2020). The elevation is another proxy for the presence of water. According to Salese et al. (2020), the basin was initially filled up to −2,243 m, which is here regarded as the reference elevation for establishing ichnological suitability of the water table level. After the breach of the crater rim, the water level dropped to −2,410 m and, during the deposition of the Jezero delta, the top of the delta had the same elevation as the bottom of the breach (Salese et al., 2020). According to Schon, Head & Fassett (2012), the base level within the palaeolake was controlled by the outlet channel and was near −2,400 m.

**Figure 17:**
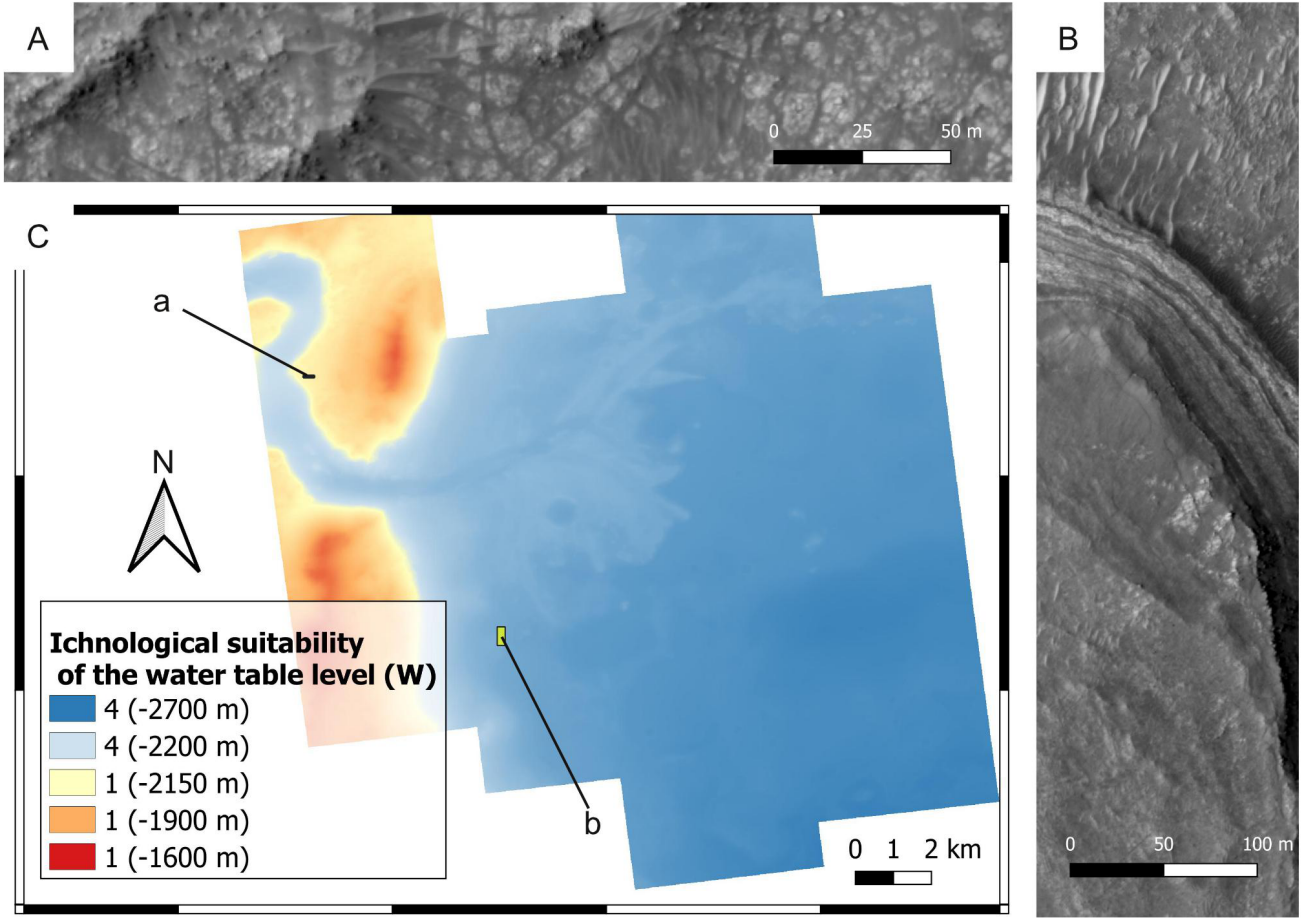
Ichnological suitability of the past water table level (*W*) within the Mars 2020 Landing Site. Quantities within parentheses refer to elevation. (A) Crater rim unit that was not submerged by the Jezero palaeolake. The outcrop exhibits rocks of the crater rim blocky unit. HiRISE image, see C for the position of the outcrop. (B) Layered facies deposited in the Jezero palaeolake. The layered deposits consist of the delta thinly layered unit, whereas the lower area comprises deposits of the crater floor fractured 2 unit. HiRISE image, see C for the position of the outcrop. (C) Suitability map of the water table level for ichnofossils. The insets labelled with a and b indicate the areas detailed in A and B, respectively.

**Suitability scoring —** Scores ranging from 1 to 4 have been attributed to the geological units of the Mars 2020 Landing Site, with 4 representing the most desirable conditions (*i.e.,* presence of a permanent water table) for ichnofossils (Fig. 17). Since the availability of water is important for ichnofossil formation (Hasiotis & Honey, 2000; Buatois & Mángano, 2011), high scores are attributed to the locations sited below the most elevated position of the Jezero shoreline (*i.e.,* −2,243 m according to (Salese et al., 2020). This high score treshold is extended to −2,200 m to include the most elevated areas that are within the fluvial channel in the Neretva Valley.

**Figure 18:**
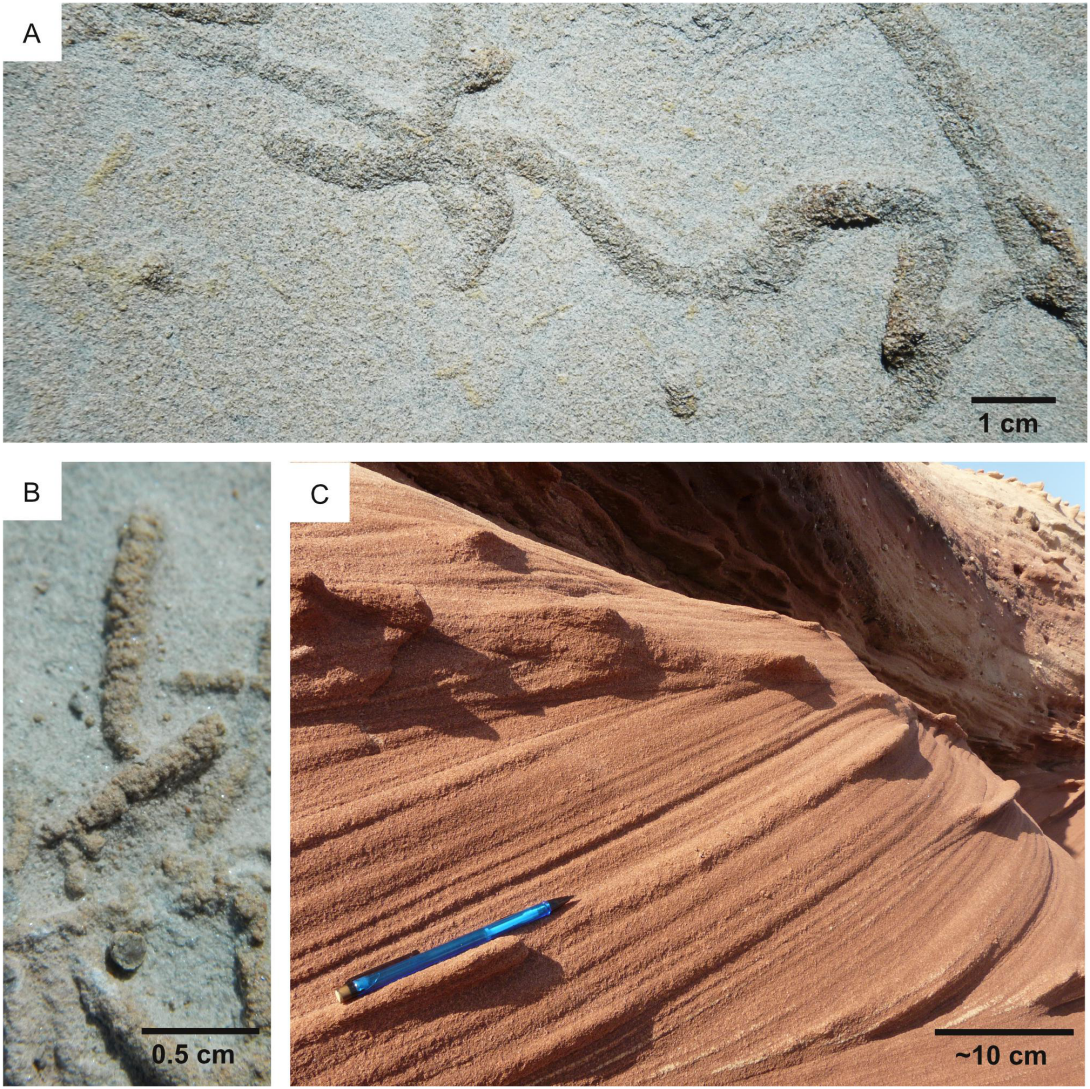
Wet *vs* hyperarid conditions. Nurra Basin (Permian-Triassic, Italy). (A) Numerous horizontal burrows (*Taenidium*) characterize the Permian units of Nurra, which deposited in a fluvial setting with permanent water table (*W* = 4). (B) Small horizontal burrows characterizing fluvial deposits (Permian) with high ichnological suitability; (C) Unbioturbated cross-stratified aeolian unit deposited in hyperarid conditions (*W* = 1).

This suitability scoring is also supported by empirical observations in the Nurra area (Permian-Triassic, Italy), where ichnofossil distribution is strongly controlled by the past water table level. Specifically, the Nurra sedimentary sequence records the transition from a fluvial ecosystem with a permanent water table (Permian) to hyper-arid conditions (Triassic) (Baucon et al., 2014). Bioturbation ichnofossils are abundant and diversified in the Permian deposits, whereas the Triassic hyper-arid deposits tend to be unbioturbated (Fig. 18). The lower Triassic hyper-arid period is documented in many other European deposits, often presenting comparable ichnological characteristics with those of the Nurra area (Durand, Meyer & Avril, 1989; Bourquin et al., 2007; Cassinis, Durand & Ronchi, 2007; Durand, 2008).

**Figure 19:**
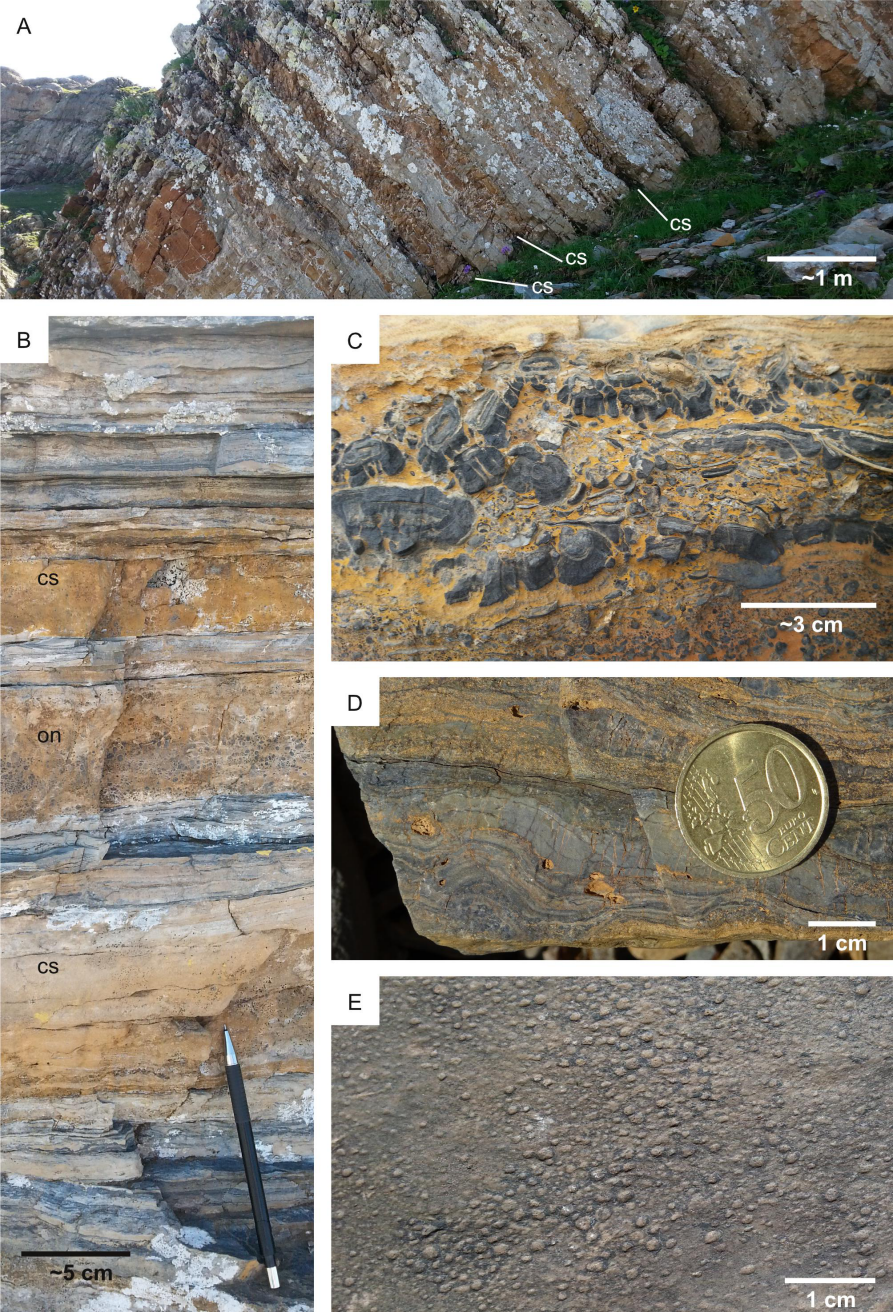
Biostratification structures in lacustrine carbonates. Collio Formation (Permian, Italy). (A) Outcrop view showing carbonate-cemented sandstone layers (cs) alternating with heterolithic fine-grained siliciclastics. (B) Oncoidal limestone (on) between two sandy layers cemented by calcite (cs), separated by fine-grained muddy layers. (C) Oncoidal bed. (D) Stromatolite. (E) Elephant skin, a microbial-induced sedimentary structure.

Even if microbial life can tolerate hyper-arid environments (Bull & Asenjo, 2013), high suitability scores for biostratification (*W* = 4) are here attributed to past aqueous environments. This is explained by the fact that increasing aridity reduces microbial diversity and abundance (Maestre et al., 2015). In addition, carbonate precipitation in aqueous environments is an excellent mechanism for biosignature preservation (Farmer & Marais, 1999; Horgan et al., 2020). This suitability scoring approach is supported by observations at the Collio Basin (Permian, Italy), which preserves lacustrine carbonates with abundant biostratification ichnofossils (Berra, Felletti & Tessarollo, 2019). In the Collio Basin, oncoids, stromatolites and MISS are associated with lacustrine palaeoenvironments dominated by carbonates, *i.e.,* spring-fed ponds at the toe of alluvial fans (Fig. 19). Similarly to the Collio Basin, the Jezero crater preserves carbonate-rich deposits in close proximity to the lake margin (Horgan et al., 2020).

## Results

Suitability for bioturbation, bioerosion and biostratification is estimated by data aggregation, that is, overlay analysis of the input layers (Table 1). Input layers map the suitability of (1) surficial cover (*K*); (2) energy regime (*E*); (3) substrate cohesiveness (*L*: for bioturbation; *H*: for bioerosion); (4) sedimentation rate (*R*); (5) position of the water table (*W*). Using Eq. (1) and the weights of Table 5, a bioturbation (A), bioerosion (B), biostratification (C) score is assigned to each location of the study area, the x y coordinates of which are indicated in subscript in the following formulas:

(2) \begin{eqnarray*}{A}_{xy}= \left( 0.4{L}_{xy}+0.2{E}_{xy}+0.3{S}_{xy}+0.1{W}_{xy} \right) - \left( 4-{K}_{xy} \right) \end{eqnarray*}

(3) \begin{eqnarray*}{B}_{xy}= \left( 0.4{H}_{xy}+0.1{E}_{xy}+0.3{S}_{xy}+0.2{W}_{xy} \right) - \left( 4-{K}_{xy} \right) \end{eqnarray*}

(4) \begin{eqnarray*}{C}_{xy}= \left( 0.1{E}_{xy}+0.4{S}_{xy}+0.5{W}_{xy} \right) - \left( 4-{K}_{xy} \right) \end{eqnarray*}

It should be noted that suitability scores associated to null weights have been omitted from the equations. The ichnological suitability scores *A*, *B*, *C* equal to 4 when the conditions of the environment (past and present) are maximally favourable for ichnosite location. This implies the absence of surficial cover overlying bedrock (*e.g.*, recent dune systems), so that the quantity 4−*K*_*xy*_ equals to 0.

**Figure 20:**
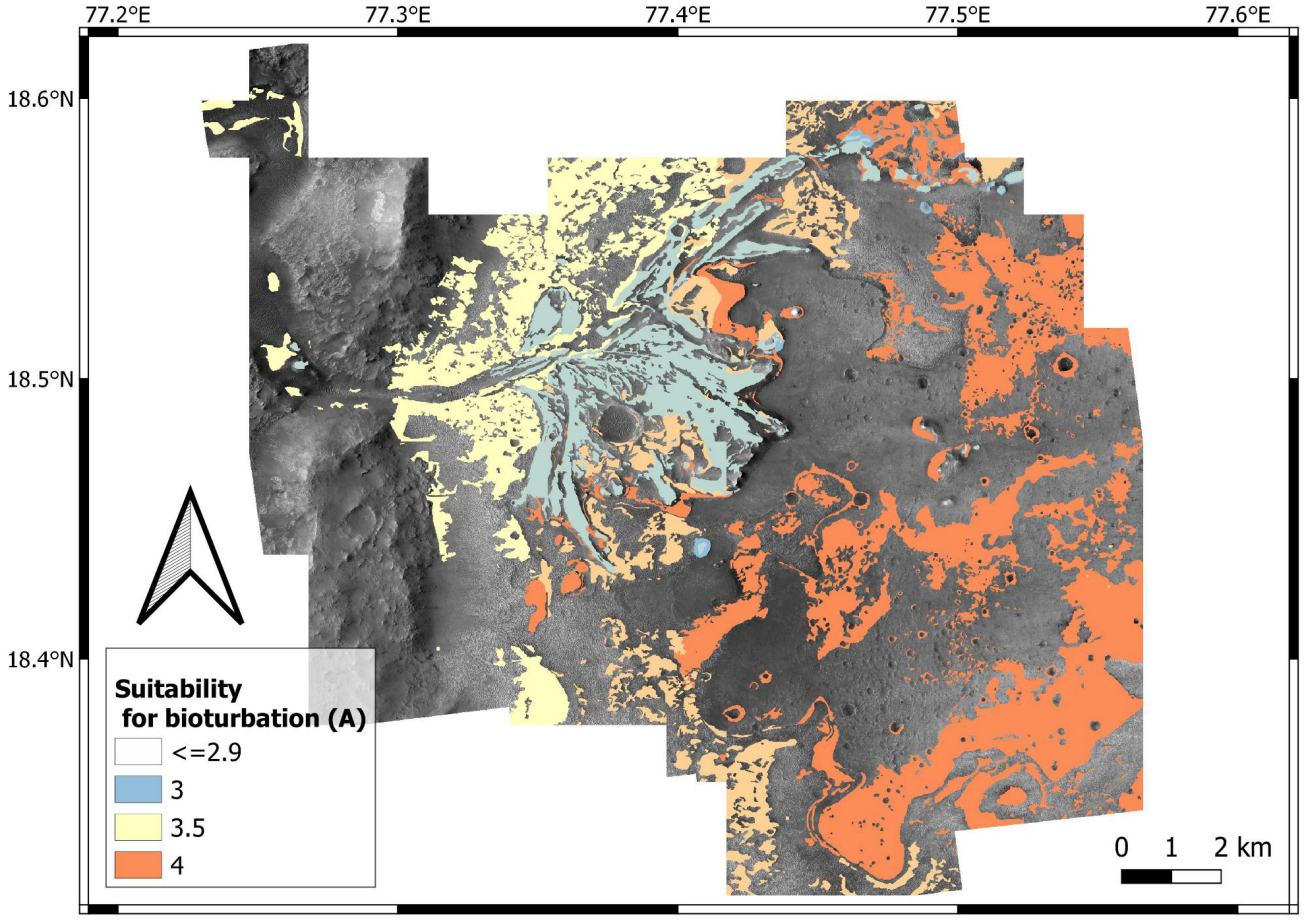
Bioturbation predictive map. The map shows threshold values (*A* ≥ 3) to identify where bioturbation ichnofossils, if any, are more likely to occur.

**Figure 21:**
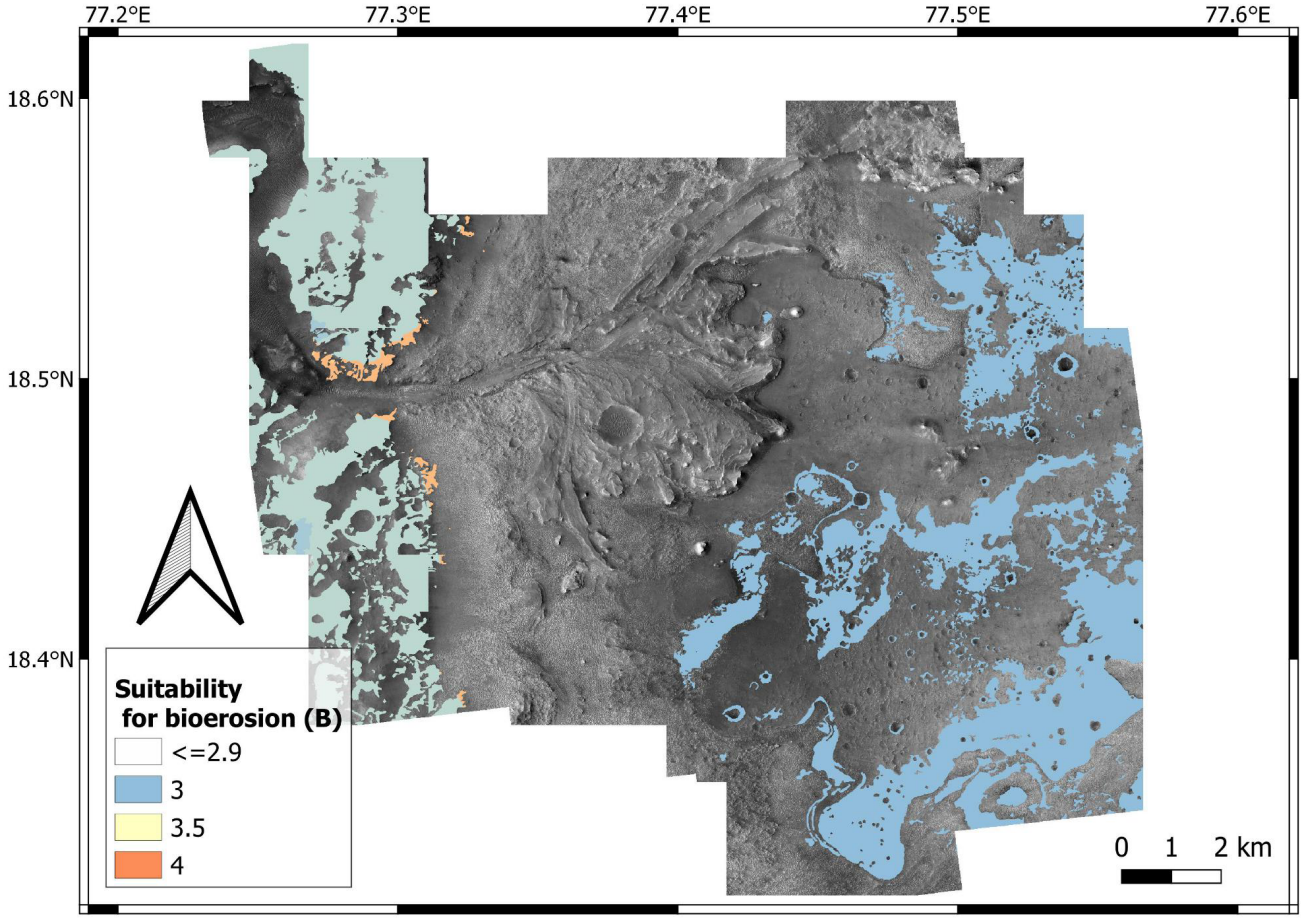
Bioerosion predictive map. The map shows threshold values (*B* ≥ 3) to identify where bioerosion ichnofossils, if any, are more likely to occur.

**Figure 22:**
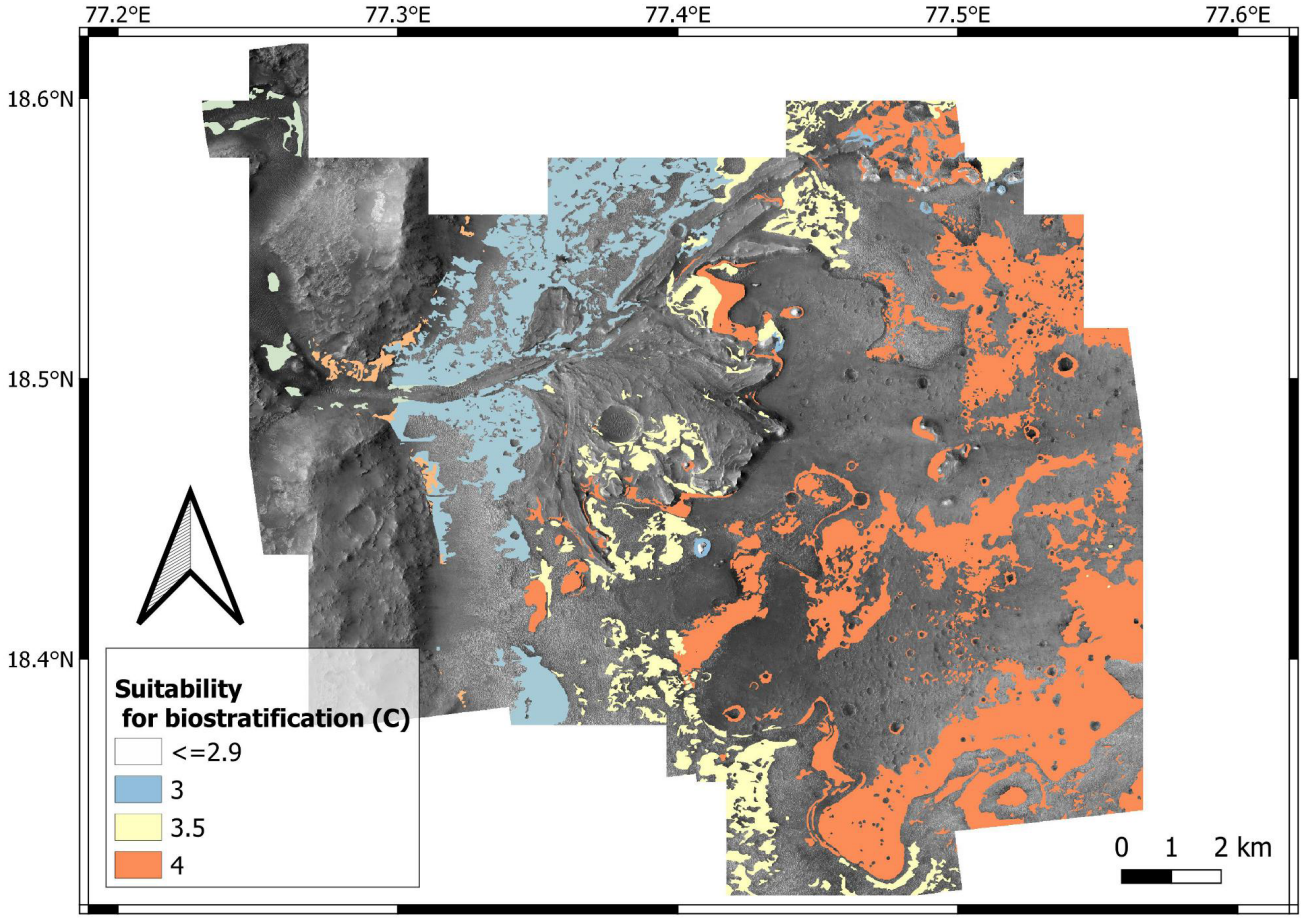
Biostratification predictive map. The map shows threshold values (C ≥ 3) to identify where biostratification ichnofossils, if any, are more likely to occur.

Equations (2)–(4) result as a set of three predictive maps (Figs. 20–22), each of which shows the suitability for a specific ichnofossil type, *e.g.*, the higher the value on the map, the more suitable the corresponding location it is for ichnofossils. Each map shows threshold values (*A* ≥ 3 for Fig. 20; *B* ≥ 3 for Fig. 21; *C* ≥ 3 for Fig. 22) to identify where ichnofossils, if any, are more likely to occur than in other locations. The value of 3 has been conventionally selected as a threshold because it splits off the highest 1/4 scores from the lowest 3/4.

## Discussion

### From ichnological neglection to ichnological appreciation

The predictive model presented in this paper identified the areas of the Mars 2020 landing site with the highest potential for ichnofossil location. This approach adds value to astrobiological research because ichnofossils have been largely neglected in the search of Martian life, with the only exception of microborings (*e.g.*, Staudigel et al., 2008; McLoughlin et al., 2010; Lepot, 2020), stromatolites (*e.g.*, Mustard et al., 2013b; Hays et al., 2017) and MISS (*e.g.*, Noffke, 2015; see the review by Baucon et al., 2017 for a more complete list of ichnological approaches to astrobiology). Bioturbation ichnofossils have completely been disregarded in the search for Martian life, with few exceptions (*e.g.*, Baucon et al., 2020a).

The neglection of ichnofossils in astrobiology plausibly derives from a common assumption, that is, the microbial nature of (eventual) Martian life. Microorganisms are regarded as the most likely candidates for a putative biota of an extraterrestrial habitat (Horneck, 2000). By contrast, ichnofossils conjure up with images of macroscopic worm burrows. It should be however noted that ichnofossils “are not the only result of the activity of burrowing or grazing macroorganisms, but also of microbes that interact with the sediment” (Noffke, 2009: p. 173). On Earth, this is not only the case of microborings but also of macroscopic bioturbation structures such as *Trichichnus*. *Trichichnus* is a macroscopic (0.1–0.7 mm in diameter) cylindrical structure deriving from bioelectrical operations resulting from bacterial activities in the oxygen-depleted part of sediments (Kȩdzierski et al., 2015). Macroscopic bioturbation ichnofossils, consisting of winding burrows, are documented from 2.1 Ga deposits of Gabon, and are tentatively attributed to the bioturbating activity of ameboid cell aggregates (El Albani et al., 2019). Tubular titanite tubes, possibly representing microbioerosion ichnofossils, are widely reported from ∼3.5 to ∼2.5 Ga metamorphosed basaltic pillow-lavas, basaltic hyaloclastite breccias and metamorphosed volcanoclastic rocks (Staudigel et al., 2008; McLoughlin et al., 2010; Lepot, 2020). 1.7 Ga deposits preserve the oldest unquestionable microboring organisms on Earth (Zhang & Golubic, 1987). Early Archaean (3.49 Ga) conical stromatolites are documented from the Pilbara Craton, Australia (Allwood et al., 2007), and even older (Nutman et al., 2016), but controversial (Allwood et al., 2018), ones are described from >3.7 Ga deposits of the Isua supracrustal belt, Greenland. Undisputed branched columnar and domal stromatolites are known from the ∼2.9 Ga Steep Rock Group in north-west Ontario (Grassineau et al., 2006). The oldest MISS have been documented in the 2.9 Ga old Pongola and Witwatersrand Supergroup, as well as the 3.2 Ga old Moodies Group, South Africa (Noffke et al., 2006; Heubeck, 2009; Noffke, 2009).

These examples show that the macroscopic record of microbial ichnofossils is abundant, spread by many geological environments, types of rocks and ages and well preserved on Earth, therefore the application of ichnology to astrobiology is much required. Although microbes are usually regarded as the most likely candidates for a putative extraterrestrial biota (Horneck, 2000), Bains & Schulze-Makuch (2016) suggest that the evolution of complex macroscopic life is nearly inevitable in any world where life has arisen and sufficient energy flux exists. This further encourages the search for extraterrestrial macroscopic ichnofossils.

### Advantages and limitations of ichnological predictive modelling

Predictive modelling has never been used to detect ichnofossils, either on Earth or beyond. To date, predictive modelling methods have successfully been used to detect archaeological artefacts (Brandt, Groenewoudt & Kvamme, 1992; Kamermans, 2004; Balla et al., 2014; Nicu, Mihu-Pintilie & Williamson, 2019) and, more recently, body fossil sites (Oheim, 2007; Anemone, Emerson & Conroy, 2011). As such, this study opens new avenues not only for the search of extraterrestrial life but also for palaeontological research on Earth.

There are multiple advantages in the application of predictive modelling to the search for ichnofossils on Mars (or other extraterrestrial locales). In parallel to the advantages of predictive modelling in archaeology (Balla et al., 2014), predictive modelling can contribute to astrobiological research by minimizing the requirements for trial observations and excavations, as it detects areas of high biological probability. On Earth, predictive modelling proved to enhance the chances of finding body fossils and archaeological artefacts (Oheim, 2007; Vaughn & Crawford, 2009), therefore, the same advantages can plausibly be expected for the search of life beyond Earth. By maximizing efforts in high probability areas, resources (time, energy, and money) used for surveying areas with little potential can be reallocated to mapping and observation efforts in higher probability areas (Vaughn & Crawford, 2009). The application of predictive modelling is particularly promising for the detection of ichnofossils. Indeed, most ichnofossils cannot be transported, with few and easily detectable exceptions (Seilacher, 2007; Buatois & Mángano, 2011). For instance, a burrow cannot be transported from delta front to prodelta settings without being destroyed by the transport processes themselves. For this reason, ichnological predictive models are particularly reliable because they mostly depend on the *in situ* characteristics of the rock record. By contrast, predictive modelling of body fossils should take into account also transport factors, *e.g.*, the body fossil of a delta front organism can be found as an allochthonous element in prodelta deposits.

The predictive modelling approach followed in this paper is not without limitations. As pointed out by other authors (Brandt, Groenewoudt & Kvamme, 1992), one weakness of the weighted approach to predictive modeling is the element of subjectivity in the weights assigned to each suitability factor. Other statistical approaches are available to deal with the subjectivity factor, *e.g.*, Bayesian statistics (Millard, 2005), machine learning (Yaworsky et al., 2020) and graph similarity analysis (Mertel, Ondrejka & Šabatová, 2018) have been applied to predictive modelling. However, there are no field data for the Jezero crater and only Earth-type life is known, therefore, application of more sophisticate statistical methods would bring the risk of overinterpretation, that is, reading too much into the limited dataset available. Fortunately, the element of subjectivity is partly reduced by the peculiarities of the ichnofossil record. Ichnofossils can be universal biosignatures, *i.e.,* they are ideally capable of detecting any type of life because they are independent from morphology, size and biochemistry of the life form they document (Baucon et al., 2017). For this reason, ichnological assumptions based on Earth-type life can readily be applied to the search of extraterrestrial ichnofossils. This universal nature of ichnofossils, and the study of reference ichnosites from different areas and geological periods, dampen the inherent subjectivity of the weighted approach to predictive modelling.

Temporal resolution is a second major limitation of the approach followed in this paper. The ichnological predictive model resulting is aimed at detecting ichnofossils formed during the presence of the Jezero palaeolake, but it is not possible to exclude *a priori* the existence of habitable conditions prior or after the Jezero palaeolake. In parallel to the future directions purported by Brandt, Groenewoudt & Kvamme (1992), a future direction for astrobiological predictive modelling is analyzing separately each geological period of Mars, thus generating an ichnological model for each period. At present this approach is not feasible because of data limitations and the urgency of focusing on the most promising subject, *i.e.*, the habitable Jezero palaeolake, where Preserverance touched down on Mars on Thursday, Feb. 18, 2021.

Spatial resolution is only an apparent limitation of the approach. Specifically, the fine resolution of the ichnological pictures (*e.g.*, Figs. 7, 9, 11) contrasts with the coarser resolution of the HiRISE-derived photogeologic map (Fig. 3) on which our model is based. Nevertheless, the resolution of the HiRISE imagery/photogeologic map does not preclude the results of our paper. In fact, HiRISE resolution allows to infer the characteristics of Mars at the scale of the depositional (sub)environment. These characteristics plausibly influenced the distribution of (eventual) life on Mars, therefore, they have a predictive value. The vast number of papers interpreting - directly or indirectly - HiRISE imagery further supports our approach (Hoefen, 2003; Ehlmann et al., 2008b; Ody et al., 2013; Goudge et al., 2015; Bramble, Mustard & Salvatore, 2017; Palumbo & Head, 2018; Rogers et al., 2018; Kremer, Mustard & Bramble, 2019; Horgan et al., 2020; Mandon et al., 2020; Stack et al., 2020). In addition, the distribution of trace fossils (fine-scale) depends upon the characteristics of the depositional environment (coarse-scale). This well-established relationship between the ichnofossil-scale and the scale of the depositional environment (see Seilacher, 2007 and references therein) strongly supports our study. By contrast, the HiRISE resolution is too coarse to spot eventual centimetric ichnofossils on Mars, but this is not the aim of this study. In other words, the approach of our paper parallels the challenge of detecting sand grains from satellite imagery of Earth. A microscope is needed to detect sand grains, but satellite imagery is enough to assess the possibility of finding sand grains on a beach.

The third limit is the lack of testing, *i.e.,* at present is not possible to evaluate the performance of the model. Predictive modelling remains useless without some form of test (Brandt, Groenewoudt & Kvamme, 1992). However, this limitation will plausibly overcome in the next years, during which the Perseverance rover will thoroughly explore the deltaic deposits of the Jezero crater. This limitation can turn out to be an advantage because the here proposed model can be used as a planning tool for organizing the path and operations of the Perseverance rover.

### An ichnological strategy for the Perseverance rover

**Figure 23:**
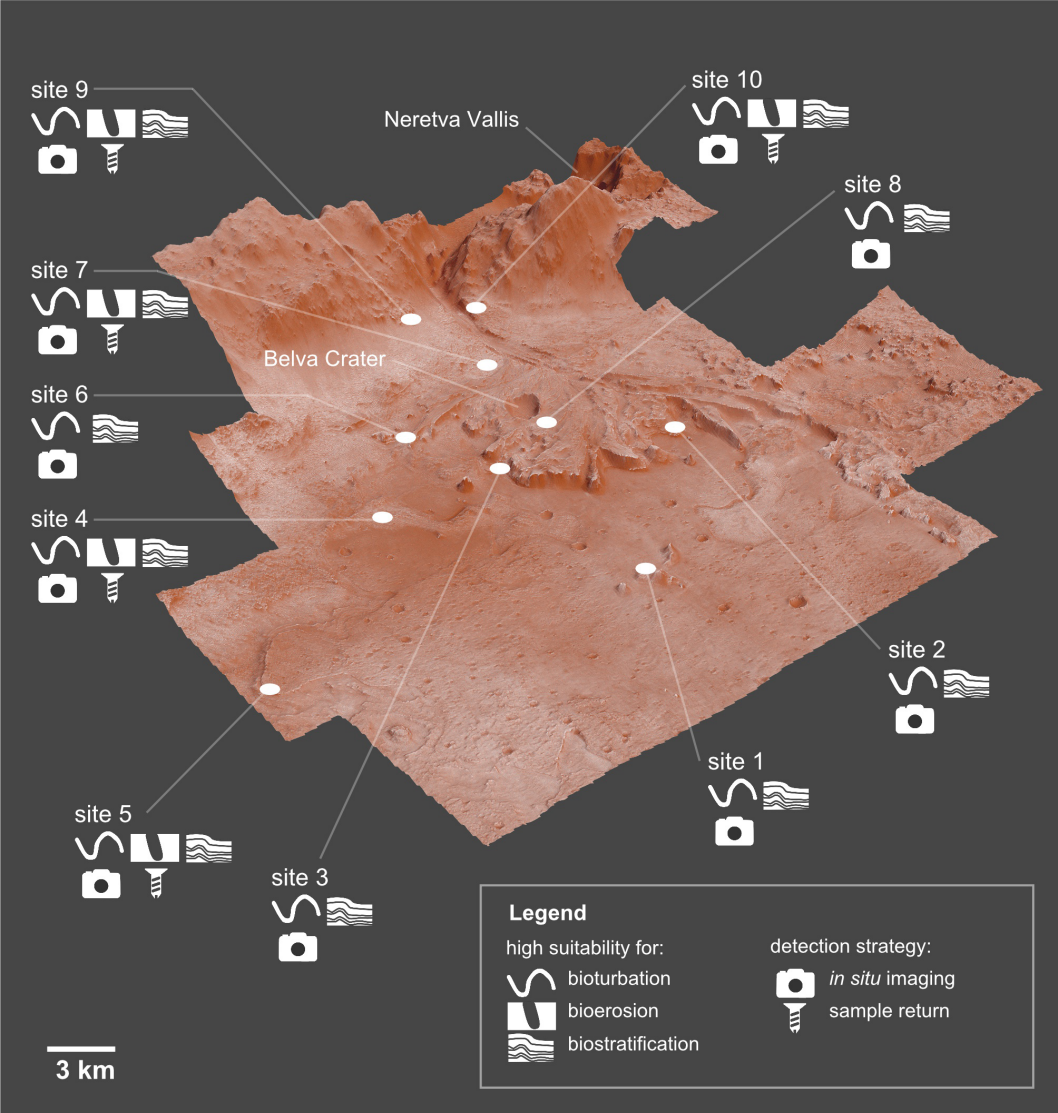
Ichnological strategy. The model indicates ten high-suitability sites and the ichnofossil types that are more likely to be present at each site, as well as the most efficient detection strategy.

Guidance in planning survey campaigns is among the major applications of predictive models on Earth (Balla et al., 2014). In parallel, the predictive maps resulting from this paper find their ideal application in suggesting a traverse plan capable of maximizing the scientific (ichnological) gain of the Perseverance rover. In other words, the predictive maps can guide or focus the Perseverance rover on the sites with the highest potential for ichnofossils. Accordingly, we identified an ichnological strategy for the Perseverance rover (Fig. 23), indicating (1) ten high-suitability sites, (2) the ichnofossil types that are more likely to be present at each site, (3) the detection strategy that is best suited for each site.

With the regard to the third aspect, visible light photography is enough to carry out an ichnological survey on Mars. Ichnofossils are morphological evidence of biological behaviour, therefore they offer the practical advantage of requiring no sophisticate tools to be detected. This said, the Perseverance rover provides the opportunity of more sophisticate analyses. The Mars 2020 mission will not only seek the signs of ancient life *in situ* but also cache a maximum of 43 samples for a possible return to Earth by a follow-on mission (Williford et al., 2018; Grady, 2020; Muirhead et al., 2020; NASA, 2021). Predictive maps can therefore suggest the optimal cache strategy, based on the assumption that each type of biogenic structure (bioturbation, bioerosion, biostratification) requires a specific type of analysis due to its size constraints. Specifically, grain size constrains the size of the smallest bioturbation and biostratification ichnofossils that may be present in a given sedimentary unit. Bioturbation and biostratification structures are formed by the displacement and reorganization of sediment grains, therefore they cannot be smaller than the smallest grain. Such size constraint does not hold for bioerosion structures, which are formed by creating an opening in hard substrata. Consequently, the imaging tools of the Perseverance rover are well suited for (potential) bioturbation and biostratification structures, but their resolution may be not enough for imaging the smallest microbioerosion structures if any are present on Mars. Imaging of microborings requires specific sample preparation techniques (*e.g.*, casting-and-embedding technique) and SEM observations (Golubic, Brent & LeCampion-Alsumard, 1970; Wisshak, 2012). Returned samples allow similar sophisticated sample preparation techniques and analytical techniques (Grady, 2020).

The first site (Fig. 23) of the ichnological traverse is located within the 7.7 × 6.6 km ellipse in which the rover landed (see Farley et al., 2020 for more detail about the landing ellipse). Site 1 displays distal delta remnants, which are suitable for both bioerosion and biostratification. Site 2 and 3 are also related to a deltaic depositional setting, although their position is more proximal with respect to site 1. Our results suggest employing the imaging tools of the Perseverance rover for the search of eventual bioturbation and biostratification ichnofossils preserved in the deltaic units. The crater floor units outcrop at sites 4 and 5, which have a high potential for bioturbation and a moderate potential for bioerosion. The possible presence of microbioerosional ichnofossils suggests collecting samples for a future sample-return mission. Site 6 displays deltaic units that are particularly promising for bioturbation, thus encouraging imaging-based observations. Close to site 6, the carbonate-rich deposits of site 7 outcrop. Here, sampling is suggested in order to better evaluate the presence of stromatolite laminations. We suggest visiting site 8, located south of the Belva crater, because here the delta truncated curvilinear layered units outcrop. These units outcrop over a relatively limited area, therefore site 8 offers one of the few excellent exposures of these units. The substrate conditions of site 9 and 10 are particularly suitable for bioerosion, for which reason sampling is suggested. These sites are located along the border between areas suitable for bioerosion and bioturbation, hence they allow to maximize scientific gain.

It should be noted that scientific gain is not the only element of traverse planning, which can be seen as an optimization process in which science gain and the level of safety are maximized while driving energy is minimized (Ono et al., 2015; Ono et al., 2020; Fink et al., 2019). Identifying and avoiding terrain hazards are particularly important aspects for the safety of planetary rovers, *e.g.*, the Spirit rover ended its mission because it got stuck in soft terrain and pointy rocks damaged the wheels of the rover Opportunity (Ono et al., 2015). The predictive maps resulting from this paper are released as raster files (Supplemental Information 2–4) and therefore they can be easily integrated in any optimization process related to traverse planning. Supplemental Materials (SM) also include a QGIS-ready zipped archive with both input and predictive layers (SM5). The raster maps provided in this paper can be combined with other information that is useful to optimize the success of the traverse, *e.g.*, terrain smoothness maps and/or maps of terrain hazards. This step is important because some of the predicted areas may be not safely accessible by the Perseverance rover.

## Conclusions

This study applied—for the first time—predictive modelling to the search of ichnofossils on Mars. The resulting predictive maps show which areas of the Mars 2020 Landing Site are more suitable for potential ichnofossils, *i.e.,* the delta remnants are particularly favourable for bioturbation ichnofossils; the crater rim is suitable for bioerosion ones; the crater margin is amenable for biostratification structures. These predictions are referred to the time in which the Jezero crater hosted a lake, but further research is needed to provide a more complete predictive picture addressing the ichnological suitability for units deposited prior and after the Jezero palaeolake. The ichnological predictive maps allowed to deliver an imaging and sampling plan capable of maximizing the scientific gain of the Perseverance rover. As such, this study provides planning tools that are useful not only for the upcoming in-situ analyses conducted by the Perseverance rover but also for the follow-up sample-return missions. Based on our research on reference ichnosites, we furthermore conclude that, if life ever existed on Mars, it presumably left traces of interaction with the substrate, preserved as bioturbation, bioerosion or biostratification structures that can be easily detected through Preserverance instruments, extending exponentially the chances of finding evidences of the (past) activity of a single life form. By contrast, the preservation of other biosignatures (body fossils, isotopic and chemical evidence) is more difficult. Also, many ichnofossils, by definition corresponding to sedimentary structures, are independent from the characteristics (morphology, biochemistry and size) of their producers, allowing to make robust predictions on the morphology and spatial/palaeoenvironmental distribution of (eventual) ichnofossils produced by extraterrestrial organisms that differ from Earth-type life. For these reasons, ichnofossils represent a promising new frontier in the search of extraterrestrial life, and predictive modelling is the ideal complementary tool for detecting their presence on Mars.

##  Supplemental Information

10.7717/peerj.11784/supp-1 Supplemental Information 1 QGIS field calculator code for relating scores to the spatial distribution of the geological units The code snippet is intended for the photogeologic map of Stack et al. (2020).

10.7717/peerj.11784/supp-2 Supplemental Information 2 Predictive layer for bioturbation (A)

10.7717/peerj.11784/supp-3 Supplemental Information 3 Predictive layer for bioerosion (B)

10.7717/peerj.11784/supp-4 Supplemental Information 4 Predictive layer for biostratification (C)

10.7717/peerj.11784/supp-5 Supplemental Information 5 GIS for the ichnological suitability of the Jezero crater, Mars A QGIS-ready project with both input and predictive layers.

## Additional Information and Declarations

### Data Availability

The following information was supplied regarding data availability: QGIS-ready shapefiles of the Jezero Crater are available in the Supplemental File 7.
