# Supplementary material for: A predictive model for the ichnological suitability of the Jezero crater, Mars: searching for fossilized traces of life-substrate interactions in the 2020 Rover Mission Landing Site
Source: PeerJ. 2021 Sep 23;9:e11784. doi: 10.7717/peerj.11784 (PMC8466086; doi:10.7717/peerj.11784)
Supplement: Supplemental Information 1 — The code snippet is intended for the photogeologic map of Stack et al. (2020). [file peerj-09-11784-s001.docx]

Manuscript submission to *PeerJ |* Type of contribution: *Article*

**Code snippets for the field calculator of QGIS**

Supplemental Material 1 for the paper entitled:

*A predictive model for the ichnological suitability of the Jezero crater, Mars: searching for fossilized traces of life-substrate interactions in the 2020 Rover Mission Landing Site*

Andrea Baucon^1,2^, Carlos Neto de Carvalho^2,3^, Antonino Briguglio^1^, Michele Piazza^1^, Fabrizio Felletti^4^

1 DISTAV, University of Genova, Genova, Italy

2 Geology Office of Idanha-a-Nova, Naturtejo UNESCO Global Geopark, Idanha-a-Nova, Portugal

3 Instituto D. Luiz, University of Lisbon. Faculdade de Ciências da Universidade de Lisboa, Lisbon, Portugal

4 Dipartimento di Scienze della Terra ‘Ardito Desio’, Milan, Italy

# Substrate suitability for bioturbation (L)

CASE WHEN BedrkUName = 'Crater floor fractured 1' THEN 4

WHEN BedrkUName = 'Crater floor fractured 2' THEN 4

WHEN BedrkUName = 'Crater floor fractured rough' THEN 4

WHEN BedrkUName = 'Margin fractured' THEN 4

WHEN BedrkUName = 'Crater rim blocky' THEN 1

WHEN BedrkUName = 'Crater rim breccia' THEN 1

WHEN BedrkUName = 'Crater rim layered' THEN 1

WHEN BedrkUName = 'Crater rim rough' THEN 1

WHEN BedrkUName = 'Crater rim rough' THEN 1

WHEN BedrkUName = 'Crater rim rough' THEN 1

WHEN BedrkUName = 'Neretva Vallis layered' THEN 4

WHEN BedrkUName = 'Nili Planum fractured' THEN 1

WHEN BedrkUName = 'Delta blocky' THEN 4

WHEN BedrkUName = 'Delta layered rough' THEN 4

WHEN BedrkUName = 'Delta thickly layered' THEN 4

WHEN BedrkUName = 'Delta thinly layered' THEN 4

WHEN BedrkUName = 'Delta truncated curvilinear layered' THEN 4

ELSE 10

END

# Substrate suitability for bioerosion (H)

CASE WHEN BedrkUName = 'Crater floor fractured 1' THEN 2

WHEN BedrkUName = 'Crater floor fractured 2' THEN 2

WHEN BedrkUName = 'Crater floor fractured rough' THEN 2

WHEN BedrkUName = 'Margin fractured' THEN 2

WHEN BedrkUName = 'Crater rim blocky' THEN 4

WHEN BedrkUName = 'Crater rim breccia' THEN 4

WHEN BedrkUName = 'Crater rim layered' THEN 4

WHEN BedrkUName = 'Crater rim rough' THEN 4

WHEN BedrkUName = 'Crater rim rough' THEN 4

WHEN BedrkUName = 'Crater rim rough' THEN 4

WHEN BedrkUName = 'Neretva Vallis layered' THEN 1

WHEN BedrkUName = 'Nili Planum fractured' THEN 4

WHEN BedrkUName = 'Delta blocky' THEN 1

WHEN BedrkUName = 'Delta layered rough' THEN 1

WHEN BedrkUName = 'Delta thickly layered' THEN 1

WHEN BedrkUName = 'Delta thinly layered' THEN 1

WHEN BedrkUName = 'Delta truncated curvilinear layered' THEN 1

ELSE 10

END

# Ichnological suitability for sedimentation rate (L)

CASE WHEN BedrkUName = 'Crater floor fractured 1' THEN 4

WHEN BedrkUName = 'Crater floor fractured 2' THEN 3

WHEN BedrkUName = 'Crater floor fractured rough' THEN 4

WHEN BedrkUName = 'Margin fractured' THEN 2

WHEN BedrkUName = 'Crater rim blocky' THEN 4

WHEN BedrkUName = 'Crater rim breccia' THEN 4

WHEN BedrkUName = 'Crater rim layered' THEN 4

WHEN BedrkUName = 'Crater rim rough' THEN 4

WHEN BedrkUName = 'Crater rim rough' THEN 4

WHEN BedrkUName = 'Crater rim rough' THEN 4

WHEN BedrkUName = 'Neretva Vallis layered' THEN 3

WHEN BedrkUName = 'Nili Planum fractured' THEN 4

WHEN BedrkUName = 'Delta blocky' THEN 1

WHEN BedrkUName = 'Delta layered rough' THEN 4

WHEN BedrkUName = 'Delta thickly layered' THEN 4

WHEN BedrkUName = 'Delta thinly layered' THEN 4

WHEN BedrkUName = 'Delta truncated curvilinear layered' THEN 3

ELSE 10

END

# Ichnological suitability of the energy regime (E)

CASE WHEN BedrkUName = 'Crater floor fractured 1' THEN 4

WHEN BedrkUName = 'Crater floor fractured 2' THEN 3

WHEN BedrkUName = 'Crater floor fractured rough' THEN 4

WHEN BedrkUName = 'Margin fractured' THEN 3

WHEN BedrkUName = 'Crater rim blocky' THEN 2

WHEN BedrkUName = 'Crater rim breccia' THEN 1

WHEN BedrkUName = 'Crater rim layered' THEN 2

WHEN BedrkUName = 'Crater rim rough' THEN 2

WHEN BedrkUName = 'Crater rim rough' THEN 2

WHEN BedrkUName = 'Crater rim rough' THEN 2

WHEN BedrkUName = 'Neretva Vallis layered' THEN 1

WHEN BedrkUName = 'Nili Planum fractured' THEN 2

WHEN BedrkUName = 'Delta blocky' THEN 2

WHEN BedrkUName = 'Delta layered rough' THEN 4

WHEN BedrkUName = 'Delta thickly layered' THEN 4

WHEN BedrkUName = 'Delta thinly layered' THEN 4

WHEN BedrkUName = 'Delta truncated curvilinear layered' THEN 3

ELSE 10

END

# Ichnological suitability of the surficial cover (K)

CASE WHEN SurfUName = 'Aeolian bedforms, large' THEN 1

WHEN SurfUName = 'Aeolian bedforms, small' THEN 2

WHEN SurfUName = 'Minor Cover Us' THEN 2

WHEN SurfUName = 'Moderate Cover Us' THEN 1

WHEN SurfUName = 'Talus' THEN 3

WHEN SurfUName = 'Undifferentiated smooth' THEN 2

ELSE 4

END
